# Antimicrobial susceptibility of *Streptococcus suis* isolated from diseased pigs, asymptomatic pigs, and human patients in Thailand

**DOI:** 10.1186/s12917-018-1732-5

**Published:** 2019-01-03

**Authors:** Suganya Yongkiettrakul, Krissana Maneerat, Buppa Arechanajan, Yuwares Malila, Potjanee Srimanote, Marcello Gottschalk, Wonnop Visessanguan

**Affiliations:** 1 National Center for Genetic Engineering and Biotechnology, National Science and Technology Development Agency, 113 Thailand Science Park, Phahonyothin Rd., Khlong Nueng, Khlong Luang, Pathum Thani, 12120 Thailand; 2 College of Alternative Medicine, Chandrakasem Rajabhat University, Bangkok, Thailand; 3 Graduate Program in Biomedical Sciences, Faculty of Allied Health Sciences, Thammasat University, Pathum Thani, Thailand; 4 Faculty of Veterinary Medicine, University of Montreal, St-Hyacinthe, QC Canada

**Keywords:** *Streptococcus suis*, Zoonosis, Bacterial meningitis, Antimicrobial susceptibility, Antimicrobial resistance, Antibiotic resistance, Multidrug resistance

## Abstract

**Background:**

Prophylaxis and treatment of emerging zoonotic *Streptococcus suis* infection in agricultural and healthcare settings mainly rely on antibiotics. However, continued use of antibiotics contributing to emergence and widespread of antibiotic resistant *S. suis* becomes a significant challenge in many endemic countries, including Thailand. Meanwhile, the knowledge of antibiotic susceptibility patterns of bacterial pathogens is required for overcoming the antimicrobial resistance problem, the information of antibiotic susceptibility of *S. suis* strains isolated in Thailand remains limited. This study aims to assess the susceptibility of Thai-isolated *S. suis* strains to different antibiotic classes in order to gain an insight into the distribution of antibiotic-resistant patterns of *S. suis* strains in different regions of Thailand.

**Results:**

This study revealed the antimicrobial resistance and multidrug resistance of 262 *S. suis* strains isolated in different regions of Thailand. Susceptibility testing indicated widespread resistance to macrolides and tetracyclines of *S. suis* strains in the country. Beta-lactam antibiotic drugs (including cefotaxime and ceftiofur), vancomycin, chloramphenicol, as well as florfenicol were potentially the most effective therapeutic drugs for the treatment of *S. suis* infection in both pigs and humans. High prevalence of intermediate susceptibility of *S. suis* isolated from asymptomatic pigs for penicillin G, gentamicin, enrofloxacin, and norfloxacin could be the premise of the emergence of *S. suis* antibiotic resistance. Resistance was also found in *S. suis* strains isolated from asymptomatic pigs indicating that they could act as reservoirs of antibiotic resistance genes.

**Conclusions:**

To the best of our knowledge, this is the first report on antimicrobial resistance of a large collection of *S. suis* strains isolated from pigs and humans in Thailand. It revealed the multidrug resistance of *S. suis* strains in pigs and humans. The information gained from this study raises an awareness and encourage best practices of appropriate antibiotic drug prescribing and use among human health and agriculture sectors.

**Electronic supplementary material:**

The online version of this article (10.1186/s12917-018-1732-5) contains supplementary material, which is available to authorized users.

## Background

*Streptococcus suis* is facultative anaerobic gram-positive α-hemolytic coccus and classified, based on cell-wall antigenic determinants, to be related to Lancefield group D streptococci. It is an important zoonotic bacterial pathogen of pigs worldwide. *S. suis* naturally colonizes upper respiratory tract of pigs, particularly the tonsils and nasal cavities [1, 2]. It can cause systemic diseases in newborn and, more commonly, weaned piglets, resulting in sepsis, meningitis, endocarditis and arthritis [3, 4]. Moreover, *S. suis* is an emerging zoonotic pathogen of humans who came in contact with infected pigs or consumed pork products that get contaminated with this pathogenic bacteria [5, 6]. Thirty-five serotypes (serotype 1–34 and serotype 1/2) of *S. suis* were originally classified based on polysaccharide capsules by using the coagglutination method [7–9]. However, recent studies, using DNA-based approaches, have shown that serotypes 20 22, 26, 33, 32, and 34 belong to novel bacterial species [10, 11]. Moreover, novel 9 capsular polysaccharide synthesis (*cps*) loci (NCLs) of non-typable *S. suis* strains have been identified based on DNA sequencing [12, 13]. Therefore, strict *S. suis* species currently comprise of 38 serotypes. Serotype 2 of *S. suis* is considered as the most common type recovered from both pigs and humans worldwide and other serotypes, such as 1, 3, 5, 7, 8, 9, 14, 16, 21 and 24, are also capable to induce disease in pigs and, some of them, also in humans [7–9, 14, 15]. To prevent or control *S. suis* infection in pigs and to deliver safer pork products for consumers, antimicrobial agents have long been applied in pig farming industries. However, the increased usage of antimicrobial agents in pigs and humans causes the antimicrobial resistance [16] that has become a global problem in recent years.

The antimicrobial agents and antibiotic classes used for prophylaxis and treatment of *S. suis* infections in pigs and humans are somewhat similar. Beta-lactam antibiotics (penicillin, ceftriaxone and ceftiofur) and fluoroquinolone antibiotics such as enrofloxacin are used in pigs and humans to treat *S. suis* infections [16–18]. Generally, three major antibiotics (penicillin, ampicillin and trimethoprim-sulfonamides) are frequently used in *S. suis* infection [16]. The increasing cases of antimicrobial resistance in *S. suis* isolated from pigs and humans have been reported from many countries in America, Asia and Europe [19, 20]. Notably, resistant *S. suis* has been identified as a reservoir for antibiotic resistance genes which can be transferred horizontally to streptococcal human pathogens such as *S. pyogenes*, *S. pneumoniae* and *S. agalactiae* [21].

Acute bacterial infection for humans and animals relies on effective antibiotic treatment. Monitoring the susceptibility pattern of bacterial pathogens to antibiotic drugs is therefore an important tool that provides an evidence-based guidance for further optimizing effective antimicrobial treatment options and following up the emergence of antibiotic drug resistance. The prevalence of antimicrobial resistance (AMR) pattern of particular pathogen is geographically variable. Hence, knowledge of the susceptibility pattern of bacterial pathogen, in certain regions is necessary for controlling the AMR problem. So far, antimicrobial susceptibility data of *S. suis* isolated in Thailand has not been well reported and available studies have focused on human cases [22–26]. Lakkitjareon et al. investigated the antimicrobial profile of 52 *S. suis* isolates from healthy pigs in Northern Thailand during 2008 to 2009 by disk diffusion susceptibility test [27]. The results showed high rate of lincomycin and tetracycline resistance but most of the isolates remained susceptible to ceftiofur, ampicillin, amoxicillin, penicillin and enrofloxacin.

The study described herein, aimed to assess the antimicrobial susceptibility of *S. suis* isolated from both human patients (epidemic and sporadic cases) and pigs (diseased and asymptomatic pigs) in northern, central, and southern regions of Thailand. The information of antimicrobial resistance of Thai-isolated *S. suis* strains could have implications for optimizing the therapeutic treatment for zoonosis and controlling the emergence of antibiotic-resistant *S. suis* in the country and worldwide.

## Results

The antimicrobial susceptibility of 262 Thai *S. suis* isolated strains was determined using 20 antibiotic drugs with different modes of inhibition. It is noted that multidrug resistance (MDR) is defined as resisting to at least three different classes of agents [32]. The result showed that there were 144 distinct antimicrobial resistance (AMR) patterns (Additional file 1: Table S1). None of Thai *S. suis* isolated strains used in this study exhibited drug susceptibility to all tested 20 antibiotic drugs. Overall, 99.3% (260/262) of Thai *S. suis* strains resisted to at least one antibiotic drug. Two out of 262 strains isolated from diseased or asymptomatic pigs shared the same antimicrobial susceptibility profile with susceptibility to 19 tested antibiotic drugs and intermediate susceptibility to norfloxacin (AMR pattern No. 1). A similar AMR pattern (AMR pattern No. 78) was observed in *S. suis* serotype 2 strains isolated from both human patients and asymptomatic pigs during 2006–2007. In addition, *S. suis* serotype 2 strains isolated from human patients and asymptomatic pigs from Northern Thailand (during 2006–2007) shared a similar AMR pattern (AMR pattern No. 79) with *S. suis* serotype 2 strains isolated from diseased pigs from central regions of Thailand (during 2012–2015).

The MDR *S. suis* strains were isolated from pigs only. Out of 235 pig-isolated *S. suis* strains, 118 strains isolated from asymptomatic pigs (118/253, 50.2%) and 20 strains isolated from diseased pigs (20/235, 8.5%) are MDR *S. suis* strains, displaying 90 different AMR patterns (Additional file 1: Table S1). Most of the MDR *S. suis* strains were AA (63 strains) and followed by non-serotype 2 (42 strains), NT (24 strains), and serotype 2 (9 strains). Two MDR *S. suis* strains, resisting to 17 out of 20 antibiotic drugs (AMR pattern No. 136) were isolated from two diseased pigs during 2006–2007. They were found in the central regions of the country where there have been intensive swine farming and production. The most predominant MDR *S. suis* strains isolated from diseased pigs resisting to AZM/CLI/DOX/ERY/GEN/TET/TIA/NOR/SXT (AMR pattern No. 97, total 12 strains) were found in different isolation periods and different regions of the country.

A total of 110 Thai *S. suis* strains, including 27 human-isolated strains, 30 strains isolated from diseased pigs, and 53 strains isolated from asymptomatic pigs, were susceptible to all 6 antibiotics drugs inhibiting cell-wall synthesis. One *S. suis* strain isolated from southern diseased pig resisted to all these 6 antibiotic drugs (AMR pattern No. 139, Additional file 1: Table S1). A total of 260 strains resisted to at least one antibiotic drug acting on protein synthesis, suggesting less effectiveness of these particular drugs for the treatment of *S. suis* infection in both human patients and pigs. Intermediate susceptibility to at least one antibiotic drug inhibiting DNA synthesis was observed for 118 Thai *S. suis* isolated strains, including 23 human-isolated strains, 24 strains isolated from diseased pigs, and 71 strains isolated from asymptomatic pigs, suggesting the emerging of antimicrobial resistance (AMR) for these antibiotic drugs.

The distribution of antimicrobial susceptibility of Thai *S. suis* isolated strains is summarized in Table 1. Thai *S. suis* isolated strains showed high level of antimicrobial susceptibility to CTX (93.1%), CTF (94.7%), VAN (96.6%), and FFC (92.4%). Susceptibility of Thai *S. suis* isolated strains to CLI (6.5%), DOX (9.2%), TET (5.0%) and TIA (2.3%) indicates the high prevalence of antibiotic-resistant *S. suis* against these drugs. Intermediate level of antibiotic susceptibility was relatively high for PEN (33.2%), GEN (23.3%), ENR (21.4%), and NOR (27.9%), suggesting the emergence of AMR problem for these antibiotic drugs in *S. suis*.

**Table 1:** Antimicrobial susceptibility of Thai *Streptococcus suis*

Antibiotic drugs	Thai-isolated *S.suis* (262 strains)					
	Susceptible		Intermediate		Resistant	
Ampicillin (AMP)	192	(73.3%)	25	(9.5%)	45	(17.2%)
Cephalexin (CFL)	165	(63.0%)	5	(1.9%)	92	(35.1%)
Cefotaxime (CTX)	244	(93.1%)	4	(1.5%)	14	(5.3%)
Ceftiofur (CTF)	248	(94.7%)	5	(1.9%)	9	(3.4%)
Penicillin G (PEN)	119	(45.4%)	87	(33.2%)	56	(21.4%)
Vancomycin (VAN)	253	(96.6%)	5	(1.9%)	4	(1.5%)
Azithromycin (AZM)	40	(15.3%)	4	(1.5%)	218	(83.2%)
Chloramphenicol (CHL)	229	(87.4%)	17	(6.5%)	16	(6.1%)
Clindamycin (CLI)	17	(6.5%)	0	(0%)	245	(93.5%)
Doxycycline (DOX)	24	(9.2%)	5	(1.9%)	233	(88.9%)
Erythromycin (ERY)	43	(16.4%)	7	(2.7%)	212	(80.9%)
Florfenicol (FFC)	242	(92.4%)	2	(0.8%)	18	(6.8%)
Gentamicin (GEN)	100	(38.2%)	61	(23.3%)	101	(38.5%)
Tetracycline (TET)	13	(5.0%)	7	(2.7%)	242	(92.4%)
Tiamulin (TIA)	6	(2.3%)	45	(17.2%)	211	(80.5%)
Ciprofloxacin (CIP)	155	(59.2%)	36	(13.7%)	71	(27.1%)
Enrofloxacin (ENR)	143	(54.6%)	56	(21.4%)	63	(24.0%)
Norfloxacin (NOR)	65	(24.8%)	73	(27.9%)	124	(47.3%)
Levofloxacin (LEV)	202	(77.1%)	6	(2.3%)	54	(20.6%)
sulfamethoxazole /Trimethoprim (SXT)	95	(36.3%)	10	(3.8%)	157	(59.9%)

The antimicrobial susceptibility of 262 Thai *S. suis* isolated strains was determined using 20 antibiotic drugs with different modes of inhibition, including beta-lactams (ampicillin, cephalexin, cefotaxime, ceftiofur, penicillin G), glycopeptide (vancomycin), aminoglycoside (gentamicin), tetracyclines (doxycycline, tetracycline), amphenicols (chloramphenicol, florfenicol), pleuromutilin (tiamulin), macrolides (azithromycin, erythromycin), lincosamide (clindamycin), fluoroquinolones (ciprofloxacin, enrofloxacin, levofloxacin), quinolone (norfloxacin), and folate inhibitors (sulfamethoxazole/trimethoprim). S: susceptible; I: intermediate; R: resistant

The distribution of antibiotic susceptibility according to the sources of *S. suis* isolation is presented in Table 2 and Additional file 2: Figure S1. The statistical analysis revealed no significant correlation between the source of bacterial isolation and susceptibility of the bacteria towards antibiotic drugs acting on protein synthesis, including AZM, CHL, DOX, and TET. The results suggested that, among those three sources, the antibiotic resistant patterns of the isolated *S. suis* were similar. On the contrary, for the other drugs, there were associations between the resistant pattern and the source of isolation.

**Table 2:** Antimicrobial susceptibility of Thai *Streptococcus suis* isolated from human patients (27 strains), diseased pigs (46 strains), and asymptomatic pigs (189 strains)

Antibiotic drugs	Human patients 27 strains			Diseased pigs 46 strains			Asymptomatic pigs 189 strains			*P*-value
	S	I	R	S	I	R	S	I	R	
AMP	27 (100%)	0 (0%)	0 (0%)	40 (86.9%)	2 (4.4%)	4 (8.7%)	125 (66.1%)	23 (12.2%)	41 (21.7%)	0.001^*^
CFL	27 (100%)	0 (0%)	0 (0%)	34 (73.9%)	1 (2.2%)	11 (23.9%)	104 (55.0%)	4 (2.1%)	81 (42.9%)	< 0.001^*^
CTX	27 (100%)	0 (0%)	0 (0%)	38 (82.6%)	2 (4.4%)	6 (13.0%)	179 (94.7%)	2 (1.1%)	8 (4.2%)	0.029^*^
CTF	27 (100%)	0 (0%)	0 (0%)	39 (84.8%)	3 (6.5%)	4 (8.7%)	182 (96.3%)	2 (1.1%)	5 (2.6%)	0.018^*^
PEN	27 (100%)	0 (0%)	0 (0%)	33 (71.7%)	8 (17.4%)	5 (10.9%)	59 (31.2%)	79 (41.8%)	51 (27.0%)	< 0.001^*^
VAN	27 (100%)	0 (0%)	0 (0%)	42 (91.3%)	1 (2.2%)	3 (6.5%)	184 (97.4%)	4 (2.1%)	1 (0.5%)	0.042^*^
AZM	5 (18.5%)	0 (0%)	22 (81.5%)	9 (19.6%)	0 (0%)	37 (80.4%)	26 (13.8%)	4 (2.1%)	159 (84.1%)	0.619
CHL	24 (88.9%)	3 (11.1%)	0 (0%)	37 (80.4%)	4 (8.7%)	5 (10.9%)	168 (88.9%)	10 (5.3%)	11 (5.8%)	0.254
CLI	5 (18.5%)	0 (0%)	22 (81.5%)	5 (10.9%)	0 (0%)	41 (89.1%)	7 (3.7%)	0 (0%)	182 (96.3%)	0.006^*^
DOX	0 (0%)	0 (0%)	27 (100%)	2 (4.4%)	2 (4.4%)	42 (91.3%)	22 (11.6%)	3 (1.6%)	164 (86.8%)	0.114
ERY	4 (14.8%)	4 (14.8%)	19 (70.4%)	9 (19.6%)	1 (2.2%)	36 (78.2%)	30 (15.9%)	2 (1.1%)	157 (83.0%)	0.001*
FFC	26 (96.3%)	1 (3.7%)	0 (0%)	36 (78.2%)	0 (0%)	10 (21.8%)	180 (95.3%)	1 (0.5%)	8 (4.2%)	< 0.001^*^
GEN	22 (81.5%)	3 (11.1%)	2 (7.4%)	11 (23.9%)	9 (19.6%)	26 (56.5%)	67 (35.5%)	49 (25.9%)	73 (38.6%)	< 0.001^*^
TET	0 (0%)	0 (0%)	27 (100%)	3 (6.5%)	2 (4.4%)	41 (89.1%)	10 (5.3%)	5 (2.6%)	174 (92.1%)	0.552
TIA	0 (0%)	23 (85.2%)	4 (14.8%)	4 (8.7%)	5 (10.9%)	37 (80.4%)	2 (1.1%)	17 (9.0%)	170 (89.9%)	< 0.001^*^
CIP	20 (74.1%)	5 (18.5%)	2 (7.4%)	32 (69.5%)	5 (10.9%)	9 (19.6%)	103 (54.5%)	26 (13.8%)	60 (31.7%)	0.048^*^
ENR	15 (55.6%)	12 (44.4%)	0 (0%)	23 (50.0%)	15 (32.6%)	8 (17.4%)	105 (55.6%)	29 (15.3%)	55 (29.1%)	< 0.001^*^
NOR	5 (18.5%)	19 (70.4%)	3 (11.1%)	13 (28.3%)	13 (28.3%)	20 (43.4%)	47 (24.9%)	41 (21.7%)	101 (53.4%)	< 0.001^*^
LEV	27 (100%)	0 (0%)	0 (0%)	41 (89.1%)	1 (2.2%)	4 (8.7%)	134 (70.9%)	5 (2.6%)	50 (26.5%)	0.003^*^
SXT	27 (100%)	0 (0%)	0 (0%)	18 (39.1%)	6 (13.0%)	22 (47.9%)	50 (26.5%)	4 (2.1%)	135 (71.4%)	< 0.001^*^

*AMP* ampicillin, *AZM* azithromycin, *CTX* cefotaxime, *CTF* ceftiofur, *CFL* cephalexin, *CHL* chloramphenicol, *CIP* ciprofloxacin, *CLI* clindamycin, *DOX* doxycycline, *ENR* enrofloxacin, *ERY* erythromycin, *FFC* florfenicol, *GEN* gentamiycin, *LEV* levofloxacin, *NOR* norfloxacin, *PEN* penicillin G, *SXT* sulfamethoxazole/trimethoprim, *TET* tetracyclin, *TIA* tiamulin, VAN: vancomycin**.** *S* susceptible; *I* intermediate; *R* resistant. The asterisk indicates statistical significance with P-value < 0.05

All 27 strains of *S. suis* isolated from human patients showed the highest level of antimicrobial susceptibility (100%) to AMP, CFL, CTX, CTF, PEN, VAN, LEV, and SXT. The data supports that these antibiotic drugs could still be effective drugs for treating *S. suis* infection in human patients. High-level sensitivity to CTX (82.6 and 94.7%), CTF (84.8 and 96.3%), and VAN (91.3 and 97.4%) were also observed in *S. suis* isolated from diseased and asymptomatic pigs. Although *S. suis* strains isolated from pigs remained highly sensitive to CTX, CTF, and VAN, their resistance against all these drugs were detected in different regions of the country, including southern regions of the country where a number of swine productions were relatively small, indicating the distribution of these antibiotic-resistant *S. suis* strains throughout the country. Among three different isolation sources, high resistance to AMP (21.7%), CFL (42.9%), PEN (27.0%), CIP (31.7%), ENR (29.1%), LEV (26.5%), and SXT (71.4%) was observed for *S. suis* strains isolated from asymptomatic pigs. In addition, this category of *S. suis* strains also showed the highest intermediate susceptible level of PEN (41.8%).

Antibiotic drug susceptibility to CHL and FFC was relatively high in pig-isolated *S. suis* strains*.* The prevalence of *S. suis* isolated from asymptomatic pigs resisting to FFC was relatively low in Thailand. This finding was consistent with a previous study in Brazil [20]. Resistance to AZM, CLI, DOX, ERY, TET, and TIA was observed from *S. suis* strains isolated from all sources. High-level resistance to CLI (89.1 and 96.3%), ERY (78.2 and 83.0%), TET (89.1 and 92.1%), and TIA (80.4 and 89.9%) in *S. suis* isolated from diseased and asymptomatic pigs were consistent with previous studies in England [33], Spain [34], France [35], Denmark [36], America [16], Brazil [20], China [37, 38], and Korea [39]. In addition, the data clearly showed that tiamulin which has a long history of use in veterinary medicine was significantly less effective for strains isolated from pigs.

The susceptibility test using fluoroquinolones, antibiotic drugs acting on DNA synthesis, demonstrated that LEV was the most effective drug against *S. suis* strains isolated from both human patients and pigs. The highest numbers of strains with intermediate susceptibility to fluoroquinolones of *S. suis* isolated from human patients and diseased pigs was observed for ENR (44.4 and 32.6%, respectively) and NOR (70.4 and 28.3%, respectively). As a preferable veterinary medicine, enrofloxacin is commonly prescribed for the treatment of streptococcal infection and also used against gram-negative bacterial infections in pigs [40]. Therefore, the observations of intermediate susceptibility to fluoroquinolones in pig-isolated *S. suis* strains in Thailand suggest that continued administration of fluoroquinolones could eventually lead to widespread of resistance to this class of compounds.

Comparison of antibiotic resistance of *S. suis* strains isolated from diseased pigs in two discrete periods (Additional file 3: Table S2 Additional file 4: Figure S2) revealed the associations between isolation period and susceptibility of *S. suis* for CFL, PEN, AZM, CHL, ERY, CIP, and ENR. The resistance to antibiotic drugs inhibiting on protein synthesis, including AZM and ERY increased in 2012–2015. The results also showed significant increases in susceptibility of Thai *S. suis* isolated strains to CFL, PEN, CIP, and ENR in 2012–2015. For fluoroquinolones, high prevalence of *S. suis* strains susceptible to NOR (100%). Nonetheless, intermediate susceptibility against LEV (21.7%) and ENR (43.5%) was observed in 2012–2015. In addition, the result showed that the susceptibility of *S. suis* against CHL was relatively high; however, the increasing cases of intermediate susceptibility could be found in the isolation year of 2012–2015. Taken together, the data suggest a tendency of reduced efficacy of these antibiotic drugs for the treatment of *S. suis* infection in the future.

The prevalence of antibiotic resistance of *S. suis* was determined according to capsular serotype of *S. suis*, including serotype 2, non-serotype 2, AA, and NT (Table 3 and Additional file 5: Figure S3). Based on statistical analysis, there were significant associations between bacterial serotypes and the susceptibility patterns towards AMP, CFL, PEN, ERY, GEN, TET, TIA, CIP, ENR, LEV, and SXT. The results showed that most of the serotype 2 *S. suis* strains were highly sensitive to antibiotic drugs acting on cell-wall synthesis, AMP (98.3%), CFL (98.3%), CTX (98.3%), CTF (98.3%), PEN (96.6%), and VAN (100%), and antibiotic drugs inhibiting DNA synthesis, CIP (79.6%), and LEV (100%). The overall data implied that serotype 2 *S. suis* strains were prone to be susceptible to more antibiotic drugs, compared to the other *S. suis* serotypes.

**Table 3:** Antimicrobial susceptibility of Thai *Streptococcus suis*, serotype 2 (59 strains), non-serotype 2 (73 strains), autoagglutinating (91 strains), and non-typable (39 strains)

Antibiotic drugs	Serotype 2 59 strains			Non-serotype 2 73 strains			Autoagglutinating 91 strains			Non-typable 39 strains			*P*-value
	S	I	R	S	I	R	S	I	R	S	I	R	
AMP	58 (98.3%)	1 (1.7%)	0 (0%)	50 (68.5%)	6 (8.2%)	17 (23.3%)	56 (61.5%)	13 (14.3%)	22 (24.2%)	28 (71.8%)	5 (12.8%)	6 (15.4%)	< 0.001^*^
CFL	58 (98.3%)	0 (0%)	1 (1.7%)	41 (56.2%)	4 (5.5%)	28 (38.3%)	46 (50.5%)	0 (0%)	45 (49.5%)	20 (51.3%)	1 2.6%)	18 (46.1%)	< 0.001^*^
CTX	58 (98.3%)	0 (0%)	1 (1.7%)	65 (89.0%)	2 (2.7%)	6 (8.3%)	88 (96.7%)	1 (1.1%)	2 (2.2%)	33 (84.6%)	1 2.6%)	5 (12.8%)	0.078
CTF	58 (98.3%)	0 (0%)	1 (1.7%)	69 (94.5%)	1 (1.4%)	3 (4.1%)	88 (96.7%)	1 (1.1%)	2 (2.2%)	33 (84.6%)	3 (7.7%)	3 (7.7%)	0.053
PEN	57 (96.6%)	1 (1.7%)	1 (1.7%)	22 (30.1%)	34 (46.6%)	17 (23.3%)	26 (28.5%)	35 (38.5%)	30 (33.0%)	14 (35.9%)	17 (43.6%)	8 (20.5%)	< 0.001^*^
VAN	59 (100%)	0 (0%)	0 (0%)	69 (94.6%)	2 (2.7%)	2 (2.7%)	90 (98.9%)	1 (1.1%)	0 (0%)	35 (89.8%)	2 (5.1%)	2 (5.1%)	0.972
AZM	5 (8.5%)	0 (0%)	54 (91.5%)	8 (11.0%)	0 (0%)	65 (89.0%)	16 (17.6%)	3 (3.3%)	72 (79.1%)	11 (28.2%)	1 (2.6%)	27 (69.2%)	0.063
CHL	53 (89.8%)	6 (10.2%)	0 (0%)	61 (83.6%)	5 (6.8%)	7 (9.6%)	83 (91.2%)	1 (1.1%)	7 (7.7%)	32 (82.1%)	5 (12.8%)	2 (5.1%)	0.032^*^
CLI	5 (8.5%)	0 (0%)	54 (91.5%)	4 (5.5%)	0 (0%)	69 (94.5%)	3 (3.3%)	0 (0%)	88 (96.7%)	5 (12.8%)	0 (0%)	34 (87.2%)	0.187
DOX	0 (0%)	0 (0%)	59 (100%)	8 (11.0%)	1 (1.4%)	64 (87.6%)	11 (12.1%)	2 (2.2%)	78 (85.7%)	5 (12.8%)	2 (5.1%)	32 (82.1%)	0.078
ERY	4 (6.8%)	5 (8.5%)	50 (84.7%)	8 (11.0%)	1 (1.4%)	64 (87.6%)	19 (20.9%)	1 (1.1%)	71 (78.0%)	12 (30.8%)	0 (0%)	27 (69.2%)	0.002^*^
FFC	52 (88.1%)	1 (1.7%)	6 (10.2%)	70 (95.9%)	0 (0%)	3 (4.1%)	85 (93.4%)	0 (0%)	6 (6.6%)	36 (92.3%)	1 (2.6%)	2 (5.1%)	0.443
GEN	33 (55.9%)	8 (13.6%)	18 (30.5%)	26 (35.6%)	17 (23.3%)	30 (41.1%)	25 (27.5%)	28 (30.7%)	38 (41.8%)	16 (41.0%)	8 (20.5%)	15 (38.5%)	0.027^*^
TET	0 (0%)	0 (0%)	59 (100%)	2 (2.7%)	3 (4.1%)	68 (93.2%)	6 (6.6%)	1 (1.1%)	84 (92.3%)	5 (12.8%)	3 (7.7%)	31 (79.5%)	0.023^*^
TIA	1 (1.7%)	30 (50.8%)	28 (47.5%)	2 (2.7%)	8 (11.0%)	63 (86.3%)	1 (1.1%)	4 (4.4%)	86 (94.5%)	2 (5.1%)	3 (7.7%)	34 (87.2%)	< 0.001^*^
CIP	47 (79.6%)	7 (11.9%)	5 (8.5%)	38 (52.1%)	12 (16.4%)	23 (31.5%)	50 (54.9%)	10 (11.0%)	31 (34.1%)	20 (51.3%)	7 (17.9%)	12 (30.8%)	0.008^*^
ENR	32 (54.2%)	27 (45.8%)	0 (0%)	38 (52.1%)	13 (17.8%)	22 (30.1%)	55 (60.4%)	9 (9.9%)	27 (29.7%)	18 (46.1%)	7 (17.9%)	14 (35.9%)	< 0.001^*^
NOR	15 (25.4%)	27 (45.8%)	17 (28.8%)	17 (23.3%)	18 (24.6%)	38 (52.1%)	21 (23.1%)	20 (22.0%)	50 (54.9%)	12 (30.8%)	8 (20.5%)	19 (48.7%)	0.203
LEV	59 (100%)	0 (0%)	0 (0%)	48 (65.8%)	4 (5.5%)	21 (28.7%)	67 (73.6%)	1 (1.1%)	23 (25.3%)	28 (71.8%)	1 (2.6%)	10 (25.6%)	< 0.001^*^
SXT	46 (78.0%)	2 (3.4%)	11 (18.6%)	22 (30.1%)	3 (4.1%)	48 (65.8%)	16 (17.6%)	2 (2.2%)	73 (86.2%)	11 (28.2%)	3 (7.7%)	25 (64.1%)	< 0.001^*^

*AMP* ampicillin, *AZM* azithromycin, *CTX* cefotaxime, *CTF* ceftiofur, *CFL* cephalexin, *CHL* chloramphenicol, *CIP* ciprofloxacin, *CLI* clindamycin, *DOX* doxycycline, *ENR* enrofloxacin, *ERY* erythromycin, *FFC* florfenicol, *GEN* gentamicin, *LEV* levofloxacin, *NOR* norfloxacin, *PEN* penicillin G, *SXT* sulfamethoxazole/trimethoprim, *TET* tetracyclin, *TIA* tiamulin, VAN: vancomycin. S: susceptible; I: intermediate; R: resistant. The asterisk indicates statistical significance with P-value < 0.05

Compared to serotype 2 *S. suis*, non-serotype 2, AA, and NT strains exhibited less sensitive to the same drugs. High frequency of intermediate susceptibility to PEN was determined in non-serotype 2 (46.6%), AA (38.5%), and NT (43.6%), but not in serotype 2 strains (1.7%) while the serotype 2 strains exhibited high frequency of intermediate susceptibility to TIA (50.8%) ENR (45.8%), and NOR (45.8%). Susceptibility to fluoroquinolones, CIP, ENR, and LEV was similar for non-serotype 2, AA, and NT *S. suis* and lower than that of serotype 2 *S. suis*.

Among antibiotic drugs inhibiting protein synthesis used in this study, a high susceptibility to CHL (82.1–91.2%) and FFC (88.1–95.9%) was observed for all serotypes; however, high-level of intermediate susceptibility to CHL (12.8%) and FFC (2.6%) was found in the NT *S. suis* strains. The result obtained from this study was also consistent with other reports on the resistance to tetracyclin and macrolide drugs of *S. suis* in pig isolates worldwide [16]. The highest percentage of resistance to AZM (69.2–91.5%), CLI (87.2–96.7%), DOX (82.1–100%), ERY (69.2–87.7%), and TET (79.5–100%) was observed for all serotypes. In addition, the percentage of resistance to AZM observed from this study was higher than reported in other countries (49% and 69% for Brazil and China, respectively) [16, 20, 38].

Among the serotypes described, serotype 2 is the most virulent and frequently isolated from both diseased pigs and human patients. Focusing on the serotype 2 *S. suis* isolated from the groups of human patients and diseased pigs (Additonal file 6: Table S3 and Additonal file 7: Figure S4), no correlation between the sources of bacterial isolation and the susceptibility patterns of *S. suis* was observed for AMP, CFL, CTX, CTF, PEN, VAN, CHL, DOX, TET, CIP, ENR, and LEV. All serotype 2 *S. suis* strains were completely sensitive to VAN and LEV and resistant to tetracyclins (DOX and TET). The susceptibility test showed that all serotype 2 *S. suis* isolated strains in Thailand remained sensitive to beta-lactams. Although most of the serotype 2 strains isolated from both human patients and diseased pigs still exhibited sensitive susceptibility towards AMP, CFL, CTX, CTF, and PEN, the cases of intermediate susceptibility or resistance against these drugs was found in the strains isolated from diseased pigs in central regions of the country in 2012–2015, raising a concern about the emerging resistance of serotype 2 *S. suis* to these drugs in the country.

Although susceptibility to CIP was relatively high in serotype 2 *S. suis*, high frequency of intermediate susceptibility to CIP was found in human-isolated serotype 2 *S. suis* strains and CIP-resistant serotype 2 *S. suis* strains were determined from pig-isolated strains. Among the serotype 2 *S. suis* strains, the results also showed that the prevalence of serotype 2 *S. suis* strains resisting to AZM, CLI, ERY, FFC, GEN, TIA, NOR, and SXT was higher in the group of pig-isolated strains. This information suggest that pigs could be a significant reservoir for antibiotic-resistant serotype 2 *S. suis*.

## Discussion

Monitoring of antimicrobial susceptibility of *S. suis* is conducted worldwide, particularly in the countries with an intensive swine production. Resistance of *S. suis* to many classes of antimicrobial agents such as lincosamides, macrolides, sulphonamides and tetracycline showed high prevalence [20]. In Northern America and European countries, the resistance of lincosamides and macrolides has been increasing both for strains isolated from pigs and humans [16, 20]. A high prevalence of tetracycline resistance was reported for *S. suis* isolates in many countries including those of North America, Asia and some from Europe [16, 19, 20]. A significant increase in tetracycline resistance was found in meningitis patients from Asia [41–43] and high prevalence of tetracycline-resistant *S. suis* isolated from pigs was clearly found in different regions of China [44]. Resistance to cephalosporin was reported in both China and Europe [45–47]. However, among common antibiotics used for the treatment of *S. suis* infection, the prevalence of *S. suis* strains resistant to penicillin (0–27%), ampicillin (0.6–23%) and ceftiofur (0–23%) was still low in many countries [20].

This study revealed the antimicrobial susceptibility of *S. suis* strains isolated in Thailand. Similar AMR patterns determined from *S. suis* strains isolated from different sources and in discrete periods of time could suggest a zoonotic transmission of AMR *S. suis* between pigs and humans and widespread of antibiotic-resistant *S. suis* across the country. The AMR patterns of Thai *S. suis* isolated strains also revealed that only *S. suis* strains isolated from pigs exhibited MDR and most of the MDR *S. suis* strains were isolated from asymptomatic pigs. This finding confirmed that asymptomatic pigs could potentially serve as reservoirs for MDR *S. suis*. As a result, a narrow spectrum of effective antibiotic drugs can be used for the treatment of *S. suis* infection in both pigs and humans.

It is important to note that *S. suis* isolates used in our study were classified by serotyping method, as described [28]. Under this circumstance, *S. suis* serotypes 22, 34 (19 and 4 strains, respectively) and all NT (39 strains) were still included for the susceptibility test and data analysis in this study. The result showed that none of the serotype 2 *S. suis* strains displayed MDR pattern and high prevalence of MDR patterns were observed for AA *S. suis* strains. The antimicrobial resistance pattern showed that serotype 22 and 34 *S. suis* strains exhibited different MDR patterns and the most MDR *S. suis* strains belonged to serotype 22. This finding suggests that precise bacterial classification methods are necessary for the AMR surveillance study of this bacterial species.

Although the findings from this study are consistent with previous literatures reporting the surveillance of *S. suis* susceptibility to beta-lactam antibiotics [16–18], high prevalence of intermediate-susceptibility *S. suis* strains against penicillin were observed and the prevalence of penicillin resistance was highest in asymptomatic pig-isolated *S. suis* strains, inferring pigs being a main reservoir for penicillin resistance of *S. suis*. Hence, a proper use of penicillin for *S. suis* infection in pigs is recommended to avoid further spread of penicillin-resistant *S. suis*.

The third-generation cephalosporin, ceftiofur has been the most effective antibiotic drug for both humans and pigs until now. Nonetheless, recent evidences from China and Europe showed the emergence of resistance to the third-generation cephalosporins [39, 41, 42]. Our study also revealed the presence of ceftiofur-resistant *S. suis* in Thailand. The evidence raise an awareness of long-term administration of this antibiotic drug inducing the spread of cephalosporins resistance in *S. suis* and therefore of the need of a surveillance of the susceptibility pattern of this zoonotic pathogen.

In this study, the periodic comparison of *S. suis* strains was performed for small number of strains that were isolated from diseased pigs only and the isolation sources of two sample groups were considerably different. Although the heatmap result demonstrated the increase of *S. suis* strains susceptible to beta-lactams in 2012–2015 (Additional file 4: Fig. S2), this finding might not markedly reflect a declining trend of AMR situation for *S. suis* in the country. To evaluate the progression of AMR situation and guide for prevention and control of AMR problem in the country, AMR surveillances of *S. suis* isolates in different regions and in consecutive years needs to be continuously carried out.

Overall, the result obtained from this study confirm that beta-lactams are the current highly-effective antibiotics whereas tetracyclines and macrolides failed to treat *S. suis* infection. Our finding also supports that chloramphenicol remains the most potent antibiotic among the protein synthesis inhibitors; however, due to toxicity of this compound, its usage has been limited in humans and prohibited to be used in food-producing animals in many countries, including Thailand.

Among Thai-isolated *S. suis* strains, a wide-range resistance to drugs acting on protein synthesis was observed in both diseased and asymptomatic pigs. Macrolides have a long history of intensive use in swine industries for prophylaxis and treatment of zoonotic streptococcal diseases [34]. Therefore, overuse and misuse of these antibiotics over many years could introduce drug resistance. In this study, the high prevalence of both AZM and ERY of *S. suis* strains observed in pig population suggests a cross-resistant mechanism of these two drugs, which needs to be further investigated.

Resistance to macrolides is mainly due to erythromycin ribosomal methylase encoded by *erm* genes or by macrolide efflux protein encoded by *mef* genes. Previous studies have identified the gene *erm*(B) associated with macrolide-lincosamide-streptogramin B (MLS_B_) resistance in *S. suis* isolated from pigs and humans [48, 49]. Recently, our preliminary data analysis of sequenced genome has shown that *erm*(B) is the most common gene found in macrolide-resistant Thai *S. suis* strains and *erm*(T) and *erm*(A) are resistant determinants of pig-isolated ERY/AZM-resistant *S. suis* strains (unpublished data). The *mef*(A) gene associated in efflux-mediated erythromycin resistance for 14- and 15-membered macrolides (known as M phenotype) and *msr*(D) encoding macrolide-efflux pump were determined in Thai *S. suis* strains (unpublished data). Nonetheless, macrolide-resistant Thai *S. suis* strains without these resistant genes were found, suggesting that other resistance mechanisms could occur and need to be further investigated.

Tetracycline resistance mechanism in *Streptococcus* species is mainly due to tetracycline-resistant ribosomal protection protein and tetracycline efflux protein, encoded by *tet* genes. In *S. suis*, *tet*(B), *tet*(40), *tet*(L), *tet*(M), *tet*(O), *tet*(W), and mosaic *tet*(O/W/32/O) have been identified [21, 50]. The *tet*(W) associates with a transposable chromosomal element and carries elements in *S. suis* isolates. Characterization of *tet*(W)-carrying elements revealed that two genetic elements, both carrying *erm*(B) besides *tet*(W), were completely different, one was almost identical to a genomic island of *S. suis* genome and another one resembling a phage that also carried other antibiotic (macrolide, aminoglycoside, and streptothricin) and heavy metal (cadmium) resistance genes [51]. A 14,741-bp unstable genetic element associated with *tet*(O/W/32/O) has been detected. This element can also carry macrolide *erm*(B) and aminoglycoside (*aadE*, *aphA*) resistance genes. In the integrated form, this unstable genetic element could be found inside an integrative and conjugative elements (ICE) which is transferable at high frequency to pathogenic *Streptococcus* species [50]. Our preliminary results, obtained from analysis of sequenced genome of tetracycline-resistant *S. suis* strains isolated from pigs and humans, have determined *tet*(M), *tet*(O), mosaic *tet*(O/W/32/O), *tet*(L), and mosaic *tet*(W/N/W) (unpublished data) which need to be further validated and their mobile genetic elements must be investigated.

Mobile genetic elements (MGEs), including ICEs, transposons, plasmids, insertion sequences, integrons, prophages, and other genomics islands, play a crucial role in dissemination of AMR determinants. Recently, comprehensive analysis of AMR-associated mobilome among *Streptococcus* species showed that several AMR genes mediating resistance to antibiotics were carried by their corresponding MGEs [52]. Among the MGEs, ICEs play a major role in bacterial adaptation and *S. suis* has high rates of ICEs. Compared to other pathogenic *Streptococcus* species, *S. suis* has higher prevalent and greater diversity of MGEs. These evidences support that *S. suis* potentially serves as MGEs reservoir for playing a key role in intra- and interspecies horizontal transfer of AMR genes to other *Streptococcus* species.

## Conclusions

The data obtained from this study support that multidrug resistance of *S. suis* strains occurs in Thailand and pigs could serve as reservoirs for the spread of antibiotic-resistant *S. suis* strains. Beta-lactam antibiotic drugs remain the most effective therapeutic drugs for the treatment of *S. suis* infection in both humans and pigs in Thailand; however, a high prevalence of intermediate susceptibility of Thai-isolated *S. suis* to different antibiotic drugs indicates a tendency for AMR problems in the future. In addition, the presence of high resistance for macrolides is raising an awareness of long-term and over use of antibiotics inducing antibiotic resistance of *S. suis*. Therefore, an appropriate and careful selection of antibiotic drug choice for prophylactic and empirical treatments of zoonotic streptococcal disease are highly recommended. To tackle the AMR problem in *S. suis*, antibiotic resistance surveillance activities in both swine industries and healthcare sector are needed to guide decisions on appropriate antibiotic use. Intensive research aiming at understanding AMR mechanism including the identification of drug-resistant biomarkers, mechanism of resistant-associated gene transfer, and development of rapid diagnostics for *S. suis* identification, are urgently needed.

## Methods

### Bacterial strains

A total of 239 strains of *S. suis* isolated from diseased pigs, healthy pigs (or so-called asymptomatic pigs), and human patients (epidemic and sporadic cases), in northern, central, and southern regions of Thailand during 2006–2007, and 23 strains of *S. suis* isolated in central regions of the country during 2012–2015 were subjected to antimicrobial susceptibility test. Diseased pigs were pigs died with clinical symptoms of septicemia and meningitis whereas asymptomatic pigs were pigs did not present any clinical signs of *S. suis* disease.

The isolation of *S. suis* has previously been described in [28]. Briefly, *S. suis* strains isolated from human patients were collected from blood and cerebrospinal fluid (CSF), prior to an outbreak (2006 to March 2007) and during the outbreak (April–May 2007). *S. suis* strains isolated from diseased pigs during 2006–2007 were collected from blood. *S. suis* strains isolated from diseased pigs during 2012–2015 were collected from lungs and mesenteric lymph nodes. *S. suis* strains isolated from asymptomatic pigs were obtained from whole tonsil swab of pigs at slaughterhouses.

Bacterial identification of all *S. suis* isolated strains used in this study were conducted using conventional biochemical tests and PCR-based approaches [28]. Serotyping of *S. suis* isolated strains was performed by coagglutination test using serotype-specific anti-sera for all 35 serotypes at the Reference Laboratory for *S. suis* Serotyping, Faculty of Veterinary Medicine, University of Montreal, Canada [28]. Characteristics of *S. suis* isolated strains used in this study are summarized in Additional file 8: Table S4. *Streptococcus pneumoniae* ATCC 49619 was used as a quality control strain for each set of antimicrobial susceptibility tests and *S. suis* strain P1/7 was used as a reference strain in this study.

### Antibiotic drugs

Twenty commercially available antibiotic drugs for veterinary and human uses, including beta-lactams (ampicillin, cephalexin, cefotaxime, ceftiofur, and penicillin G), glycopeptide (vancomycin), aminoglycoside (gentamicin), tetracyclines (doxycycline, tetracycline), phenicols (chloramphenicol and florfenicol), pleuromutilin (tiamulin), macrolides (azithromycin and erythromycin), lincosamide (clindamycin), fluoroquinolones (ciprofloxacin, enrofloxacin, and levofloxacin), quinolone (norfloxacin), and folate inhibitors (sulfamethoxazole/trimethoprim) were applied for susceptibility test of *S. suis*. The antibiotic disks were purchased from Oxoid Limited (Hampshire, England). Tiamulin disk (30 μg/disk) was prepared by applying 5 μL of 6 mg/mL of tiamulin on a sterile paper disk (Oxiod disks). Antibiotic drugs used in this study classified according to mode of drug action are listed in Additional file 9: data, Table S5.

### Antimicrobial susceptibility test

To assess the antibiotic susceptibility profile of *S. suis* strains isolated from Thailand, the antibiotic susceptibility test was carried out by disk diffusion method according to a standard protocol of Clinical and Laboratory Standards Institute [29]. *S. suis* was grown overnight on Columbia agar (Sisco Research Laboratories, New Mumbai, India) supplemented with 5% defibrinated sheep blood at 37 °C in 5% CO_2_. Subsequently, colonies from the overnight culture were selected and suspended in Todd Hewitt broth (Oxoid Limited, Hampshire, England). The bacterial cell suspension was adjusted to be a 0.5 McFarland standard, equivalent to 10^6^ colony-forming units per milliliter (cfu/mL). The adjusted cell suspension was spread on 4-mm depth Mueller Hinton agar supplemented with 5% defibrinated sheep blood. The disks containing standardized known amount of antibiotic agent were placed on the bacterial agar plate. Approximately, 5–6 disks were placed per plate using a disk dispenser (BioRad, Hercules, California USA). The plates were then incubated at 37 °C in 5% CO_2_ for 18 h. During the plate incubation, the antibiotic agents diffused around the disk and inhibited the growth of bacteria, generating a clear zone known as “zone of inhibition”.

The diameter of inhibition zone of *S. suis* strains, control strain, and reference strain was measured and interpreted as susceptible (S), intermediate (I), or resistant (R), according to CLSI supplement M100S [29] for cefotaxime (CTX), azithromycin (AZM), chloramphenicol (CHL), clindamycin (CLI), doxycycline (DOX), erythromycin (ERY), tetracycline (TET), levofloxacin (LEV), and sulfamethoxazole/trimethoprim (SXT). The inhibition zone for ceftiofur (CTF), florfenicol (FFC), ciprofloxacin (CIP), enrofloxacin (ENR) and norfloxacin (NOR) was interpreted according to Soares TCS., et al. 2014 [20]. The diameter breakpoint for ampicillin (AMP), cephalexin (CFL), penicillin G (PEN), vancomycin (VAN), gentamicin (GEN), tiamulin (TIA) was taken from EUCAST and CLSI-potency Neo-Sensitabs™ User’s Guide [30] (Additional file 9: Table S5).

### Statistical analysis

The Pearson’s Chi-square (χ^2^) test was performed to determine the independence between antibiotic susceptibility and the four categorical variables of interest, including bacterial serotype, source of bacterial isolation, health status of the source, and year of isolation. The null hypothesis was stated as no association between antibiotic susceptibility and the testing categorical variable whereas the alternative hypothesis was that the susceptibility of each testing antibiotic drug was significantly associated with the testing variables. The Chi-square formula is shown as followed.

$$ {x}^2=\sum \limits_{i,j}\frac{{\left({f}_{ij}-{e}_{ij}\right)}^2}{e_{ij}} $$

where f_ij_ is the observed frequency count of events belonging to both i^th^ of category X and j^th^ of category Y and e_ij_ is the corresponding expected count if X and Y are independent. Antibiotic susceptibility (category Y) was denoted as “sensitive” (S), “intermediate sensitive” (I) and “resistance” (R). For each category X, bacterial serotype included “serotype 2”, “non-serotype 2”, “autoagglutinating (AA)”, and “non-typable (NT)”; source of bacterial isolation comprises “human patients”, “diseased pigs”, and “asymptomatic pigs”; health status of source consisted of “diseased pigs” and “asymptomatic pigs”; year of isolation was defined as the period between “2006-2007” and “2012-2015”.

The analysis was performed using function chisq.test of R package version 3.4.3 [31]. The null hypothesis of the independence assumption is to be rejected if the *P*-value of the Chi-squared test was less than a given significance level α = 0.05 (*P*-value < 0.05).

## Additional files

Additional file 1: **Table S1.** Antimicrobial resistance patterns of Thai *S. suis* isolated strains. AMP: ampicillin, AZM: azithromycin, CTX: cefotaxime, CTF: ceftiofur, CFL: cephalexin, CHL: chloramphenicol, CIP: ciprofloxacin, CLI: clindamycin, DOX: doxycycline, ENR: enrofloxacin, ERY: erythromycin, FFC: florfenicol, GEN: gentamicin, LEV: levofloxacin, NOR: norfloxacin, PEN: penicillin G, SXT: sulfamethoxazole/trimethoprim, TET: tetracyclin, TIA: tiamulin, VAN: vancomycin. *S. suis* strains isolated from human patients, diseased pigs during 2006–2007 and 2012–2015 were named as Hxxx, DP6xxx and DP15xx, respectively, when x was the identification number. (TIF 602 kb)

Additional file 2: **Figure S1.** Heatmap illustrates susceptibility, i.e. susceptible, intermediate, and resistant, of *Streptococcus suis* isolated from human patients (*n* = 27), diseased pigs (*n* = 46) and asymptomatic pigs (*n* = 189) grouped towards testing antibiotic drugs. The isolated bacteria were clustered based on the isolation sources. Associations between source of isolation and susceptibility of each antibiotic drug were analyzed using Pearson’s Chi-square dependent test. The asterisk indicates that null hypothesis of the Chi-square test was rejected (*P*-value <  0.05), suggesting a significant association. (TIF 268 kb)

Additional file 3: **Table S2.** Antimicrobial susceptibility of Thai *Streptococcus suis* strains isolated from diseased pigs during 2006–2007 and 2012–2015. During 2006–2007, the *S. suis* strains were isolated from different regions of the country (northern, 11 strains; central, 6 strains, and southern, 6 strains). All of the *S. suis* strains isolated during 2012–2015 (23 strains) were collected from central regions of the country. AMP: ampicillin, AZM: azithromycin, CTX: cefotaxime, CTF: ceftiofur, CFL: cephalexin, CHL: chloramphenicol, CIP: ciprofloxacin, CLI: clindamycin, DOX: doxycycline, ENR: enrofloxacin, ERY: erythromycin, FFC: florfenicol, GEN: gentamicin, LEV: levofloxacin, NOR: norfloxacin, PEN: penicillin G, SXT: sulfamethoxazole/trimethoprim, TET: tetracyclin, TIA: tiamulin, VAN: vancomycin. S: susceptible; I: intermediate; R: resistant. The asterisk indicates statistical significance with *P*-value < 0.05. (TIF 597 kb)

Additional file 4: **Figure S2.** Heatmap illustrates susceptibility, i.e. susceptible, intermediate, and resistant, of *Streptococcus suis*, isolated from diseased pigs during the two different periods of time towards testing antibiotic drugs. The isolated bacteria were clustered, based on the period of isolation, i.e. 2006–2007 (*n* = 23) and 2012–2015 (n = 23). Associations between the period of isolation and susceptibility of each antibiotic drug were analyzed using Pearson’s Chi-square dependent test. The asterisk indicates that null hypothesis of the Chi-square test was rejected (*P*-value <  0.05), suggesting a significant association. (TIF 244 kb)

Additional file 5: **Figure S3.** Heatmap illustrates susceptibility, i.e. susceptible, intermediate, and resistant, of *Streptococcus suis*, grouped based on serotypes, towards testing antibiotic drugs. The isolated bacteria were clustered into four serotypes, including serotype 2 (*n* = 59), non-serotype 2 (*n* = 73), autoagglutinating (*n* = 91) and non-typable (*n* = 39). Associations between source of isolation and susceptibility of each antibiotic drug were analyzed using Pearson’s Chi-square dependent test. The asterisk indicates that null hypothesis of the Chi-square test was rejected (*P*-value <  0.05), suggesting a significant association. (DOCX 70 kb)

Additional file 6: **Table S3.** Antimicrobial susceptibility of Thai serotype 2 *Streptococcus suis*. Human patients (27 strains) and pigs (32 strains), including asymptomatic (7 strains) and diseased pigs (25 strains). AMP: ampicillin, AZM: azithromycin, CTX: cefotaxime, CTF: ceftiofur, CFL: cephalexin, CHL: chloramphenicol, CIP: ciprofloxacin, CLI: clindamycin, DOX: doxycycline, ENR: enrofloxacin, ERY: erythromycin, FFC: florfenicol, GEN: gentamicin, LEV: levofloxacin, NOR: norfloxacin, PEN: penicillin G, SXT: sulfamethoxazole/trimethoprim, TET: tetracyclin, TIA: tiamulin, VAN: vancomycin. S: susceptible; I: intermediate; R: resistant. The asterisk indicates statistical significance with *P*-value < 0.05. (DOC 3450 kb)

Additional file 7: **Figure S4.** Heatmap illustrates susceptibility, i.e. susceptible, intermediate, and resistant, of serotype 2 *Streptococcus suis*, isolated from human patients and pigs, towards testing antibiotic drugs. The isolated bacteria were clustered based on host, i.e. human patients (*n* = 27) and pigs (*n* = 32) including asymptomatic and diseased pigs. Associations between source of isolation and susceptibility of each antibiotic drug were analyzed using Pearson’s Chi-square dependent test. The asterisk indicates that null hypothesis of the Chi-square test was rejected (*P*-value <  0.05), suggesting a significant association. (DOC 3449 kb)

Additional file 8: **Table S4.** Source of *Streptococcus suis* isolated strains and numbers of strains used in this study. Thai *S. suis* strains used in this study were isolated during 2006–2007, except 23 strains of serotype 2 *S. suis* obtained from central regions #2 (Nakhon Pathom) were isolated during 2012–2015. Non-serotype 2 *S. suis* isolated from diseased pigs includes serotype 1 (1 strain), 14 (1 strain), 16 (1 strain), 22 (2 strains), 23 (1 strain), 25 (1 strain), and 34 (1 strain). Non-serotype 2 *S. suis* isolated from asymptomatic pigs includes serotypes 1 (1 strain), 3 (5 strains), 5 (3strains), 7 (2 strains), 9 (7 strains), 12 (2 strains), 15 (1 strain), 16 (5 strains), 19 (2 strains), 21 (1 strain), 22 (17 strains), 24 (2strains), 25 (1 strain), 27 (1 strain), 28 (2 strains), 29(4 strains), 30 (6 strains), and 34 (3 strains). (DOC 3934 kb)

Additional file 9: **Table S5.** Inhibition zone diameter and zone interpretation of antibiotic drugs. Antibiotic drugs used in this study were classified into four different modes of inhibition including cell-wall synthesis inhibitors (6 drugs), protein synthesis inhibitors (9 drugs), DNA synthesis inhibitors (4 drugs), and antimetabolite (1 drug). The zone of inhibition was interpreted as susceptible (S), intermediate (I), or resistant (R), according to a standard protocol of Clinical & Laboratory Standards Institute (CLSI), European Committee on Antimicrobial Susceptibility Testing (EUCAST) and Ref. [20]. The interpretation according to EUCAST and CLSI 2013 was used for veterinary practice, described in EUCAST and CLSI-potency Neo-Sensitabs™ User’s Guide 2013, rev. date 11-04-2013 [30]. (DOC 3973 kb)

## Abbreviations

AA: Autoagglutinating
AMP: Ampicillin
AMR: Antimicrobial resistance
ATCC: American Type Culture Collection
AZM: Azithromycin
CFL: Cephalexin
CFU: Colony-forming unit
CHL: Chloramphenicol
CIP: Ciprofloxacin
CLI: Clindamycin
CO_2_: Carbon dioxide
CSF: Cerebrospinal fluid
CTF: Ceftiofur
CTX: Cefotaxime
DOX: Doxycycline
ENR: Enrofloxacin
ERY: Erythromycin
FFC: Florfenicol
GEN: Gentamicin
hr.(s): Hour(s)
LEV: Levofloxacin
MDR: Multidrug resistance
NOR: Norfloxacin
NT: Non-typable
PCR: Polymerase chain reaction
PEN: Penicillin G
*S. suis*: *Streptococcus suis*
SXT: Sulfamethoxazole/trimethoprim
TET: Tetracyclin
TIA: Tiamulin
VAN: Vancomycin
χ^2^: Chi-square
