# Supplementary figures and images for: Antimicrobial susceptibility of Streptococcus suis isolated from diseased pigs, asymptomatic pigs, and human patients in Thailand
Source: BMC Vet Res. 2019 Jan 3;15:5. doi: 10.1186/s12917-018-1732-5 (PMC6318959; doi:10.1186/s12917-018-1732-5)

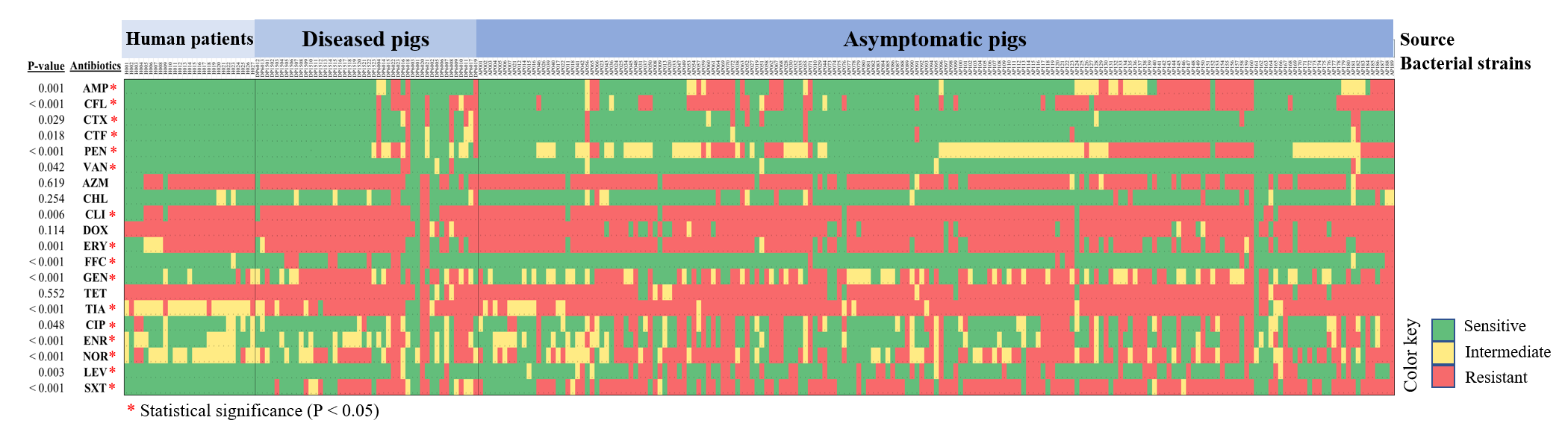

Supplement: Supplementary file 1 — Table S1. Antimicrobial resistance patterns of Thai S. suis isolated strains. AMP: ampicillin, AZM: azithromycin, CTX: cefotaxime, CTF: ceftiofur, CFL: cephalexin, CHL: chloramphenicol, CIP: ciprofloxacin, CLI: clindamycin, DOX: doxycycline, ENR: enrofloxacin, ERY: erythromycin, FFC: florfenicol, GEN: gentamicin, LEV: levofloxacin, NOR: norfloxacin, PEN: penicillin G, SXT: sulfamethoxazole/trimethoprim, TET: tetracyclin, TIA: tiamulin, VAN: vancomycin. S. suis strains isolated from human patients, diseased pigs during 2006–2007 and 2012–2015 were named as Hxxx, DP6xxx and DP15xx, respectively, when x was the identification number. (TIF 602 kb) [file 12917_2018_1732_MOESM1_ESM.tif]

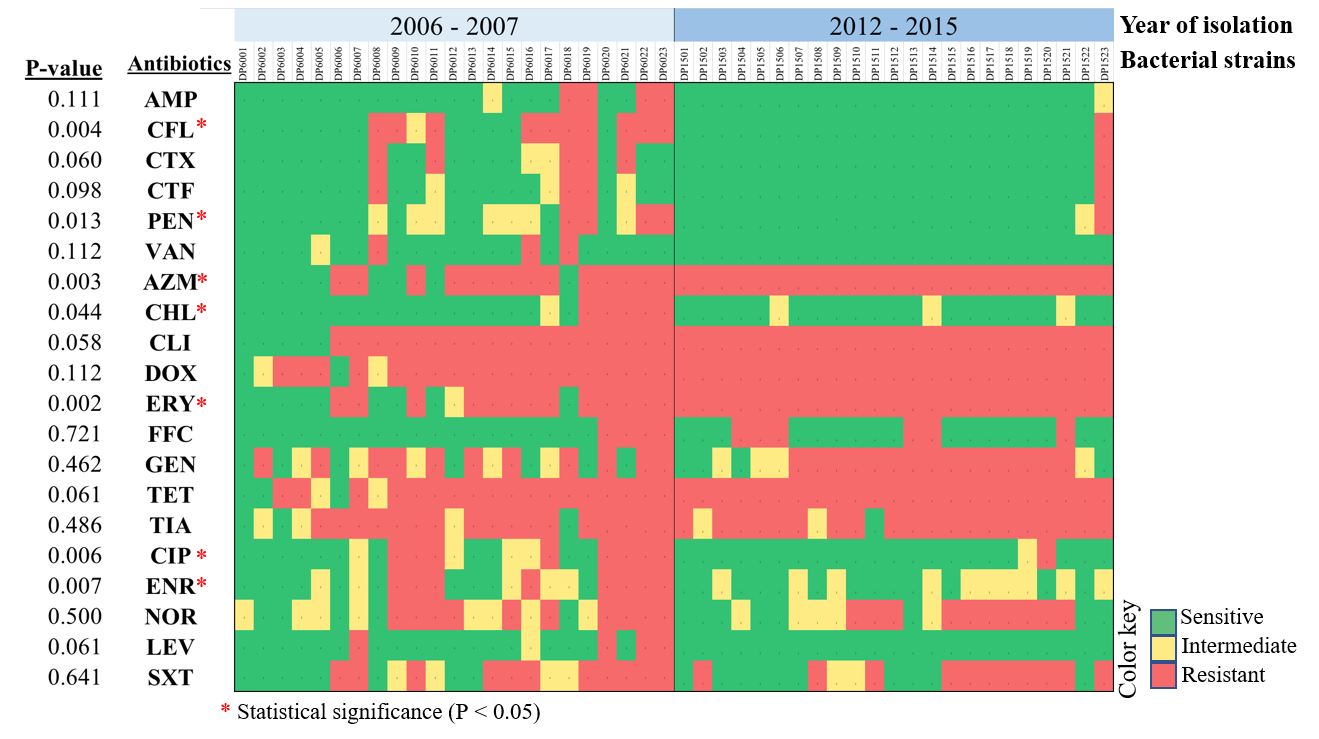

Supplement: Supplementary file 2 — Figure S1. Heatmap illustrates susceptibility, i.e. susceptible, intermediate, and resistant, of Streptococcus suis isolated from human patients (n = 27), diseased pigs (n = 46) and asymptomatic pigs (n = 189) grouped towards testing antibiotic drugs. The isolated bacteria were clustered based on the isolation sources. Associations between source of isolation and susceptibility of each antibiotic drug were analyzed using Pearson’s Chi-square dependent test. The asterisk indicates that null hypothesis of the Chi-square test was rejected (P-value < 0.05), suggesting a significant association. (TIF 268 kb) [file 12917_2018_1732_MOESM2_ESM.tif]

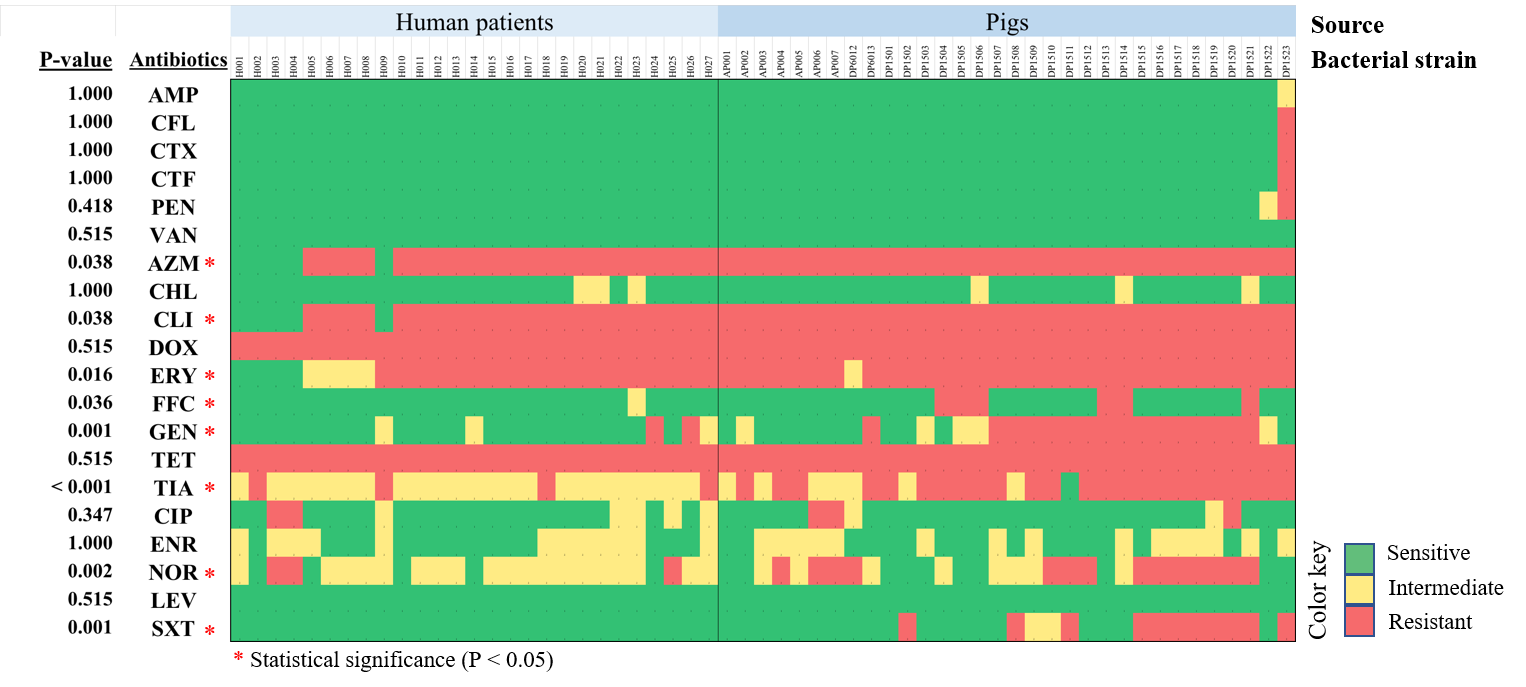

Supplement: Supplementary file 4 — Figure S2. Heatmap illustrates susceptibility, i.e. susceptible, intermediate, and resistant, of Streptococcus suis, isolated from diseased pigs during the two different periods of time towards testing antibiotic drugs. The isolated bacteria were clustered, based on the period of isolation, i.e. 2006–2007 (n = 23) and 2012–2015 (n = 23). Associations between the period of isolation and susceptibility of each antibiotic drug were analyzed using Pearson’s Chi-square dependent test. The asterisk indicates that null hypothesis of the Chi-square test was rejected (P-value < 0.05), suggesting a significant association. (TIF 244 kb) [file 12917_2018_1732_MOESM4_ESM.tif]
