# Supplementary material for: Antimicrobial susceptibility of Streptococcus suis isolated from diseased pigs, asymptomatic pigs, and human patients in Thailand
Source: BMC Vet Res. 2019 Jan 3;15:5. doi: 10.1186/s12917-018-1732-5 (PMC6318959; doi:10.1186/s12917-018-1732-5)
Supplement: Supplementary file 3 — Table S2. Antimicrobial susceptibility of Thai Streptococcus suis strains isolated from diseased pigs during 2006–2007 and 2012–2015. During 2006–2007, the S. suis strains were isolated from different regions of the country (northern, 11 strains; central, 6 strains, and southern, 6 strains). All of the S. suis strains isolated during 2012–2015 (23 strains) were collected from central regions of the country. AMP: ampicillin, AZM: azithromycin, CTX: cefotaxime, CTF: ceftiofur, CFL: cephalexin, CHL: chloramphenicol, CIP: ciprofloxacin, CLI: clindamycin, DOX: doxycycline, ENR: enrofloxacin, ERY: erythromycin, FFC: florfenicol, GEN: gentamicin, LEV: levofloxacin, NOR: norfloxacin, PEN: penicillin G, SXT: sulfamethoxazole/trimethoprim, TET: tetracyclin, TIA: tiamulin, VAN: vancomycin. S: susceptible; I: intermediate; R: resistant. The asterisk indicates statistical significance with P-value < 0.05. (TIF 597 kb) [file 12917_2018_1732_MOESM3_ESM.doc]

**Table S2**:

| **Antibiotic drugs** | **Diseased pigs** | | | | | | **P-value** |
| --- | --- | --- | --- | --- | --- | --- | --- |
|  | **Year: 2006-2007**  **23 strains** | | | **Year 2012-2015**  **23 strains** | | |  |
|  | **S** | **I** | **R** | **S** | **I** | **R** |  |
| AMP | 18 (78.3%) | 1 (4.3%) | 4 (17.4%) | 22 (95.7%) | 1 (4.3%) | 0 (0%) | 0.111 |
| CFL | 12 (52.2%) | 1 (4.3%) | 10 (43.5%) | 22 (95.7%) | 0 (0%) | 1 (4.3%) | 0.004^*^ |
| CTX | 16 (69.6%) | 2 (8.7%) | 5 (21.7%) | 22 (95.7%) | 0 (0%) | 1 (4.3%) | 0.060 |
| CTF | 17 (74.0%) | 3 (13.0%) | 3 (13.0%) | 22 (95.7%) | 0 (0%) | 1 (4.3%) | 0.098 |
| PEN | 12 (52.2%) | 7 (30.4%) | 4 (17.4%) | 21 (91.3%) | 1 (4.3%) | 1 (4.3%) | 0.013^*^ |
| VAN | 19 (82.7%) | 1 (4.3%) | 3 (13.0%) | 23 (100%) | 0 (0%) | 0 (0%) | 0.112 |
| AZM | 9 (39.1%) | 0 (0%) | 14 (60.9%) | 0 (0%) | 0 (0%) | 23 (100%) | 0.003^*^ |
| CHL | 17 (74.0%) | 1 (4.3%) | 5 (21.7%) | 20 (87%) | 3 (13%) | 0 (0%) | 0.044^*^ |
| CLI | 5 (21.7%) | 0 (0%) | 18 (78.3%) | 0 (0%) | 0 (0%) | 23 (100%) | 0.058 |
| DOX | 2 (8.7%) | 2 (8.7%) | 19 (82.6%) | 0 (0%) | 0 (0%) | 23 (100%) | 0.112 |
| ERY | 9 (39.1%) | 1 (4.3%) | 13 (56.6%) | 0 (0%) | 0 (0%) | 23 (100%) | 0.002^*^ |
| FFC | 19 (82.6%) | 0 (0%) | 4 (17.4%) | 17 (73.9%) | 0 (0%) | 6 (26.1%) | 0.721 |
| GEN | 7 (30.4%) | 5 (21.8%) | 11 (47.8%) | 4 (17.4%) | 4 (17.4%) | 15 (65.2%) | 0.462 |
| TET | 3 (13.0%) | 2 (8.7%) | 18 (78.3%) | 0 (0%) | 0 (0%) | 23 (100%) | 0.061 |
| TIA | 3 (13.0%) | 3 (13.0%) | 17 (74.0%) | 1 (4.3%) | 2 (8.7%) | 20 (87%) | 0.486 |
| CIP | 11 (47.8%) | 4 (17.4%) | 8 (34.8%) | 21 (91.3%) | 1 (4.3%) | 1 (4.3%) | 0.006^*^ |
| ENR | 10 (43.5%) | 5 (21.7%) | 8 (34.8%) | 13 (56.5%) | 10 (43.5%) | 0 (0%) | 0.007^*^ |
| NOR | 5 (21.7%) | 8 (34.8%) | 10 (43.5%) | 23 (100%) | 0 (0%) | 0 (0%) | 0.500 |
| LEV | 18 (78.3%) | 1 (4.3%) | 4 (17.4%) | 8 (34.8%) | 5 (21.7%) | 10 (43.5%) | 0.061 |
| SXT | 8 (34.8%) | 4 (17.4%) | 11 (47.8%) | 10 (43.5%) | 2 (8.7%) | 11 (47.8%) | 0.641 |
