# Supplementary material for: Antimicrobial susceptibility of Streptococcus suis isolated from diseased pigs, asymptomatic pigs, and human patients in Thailand
Source: BMC Vet Res. 2019 Jan 3;15:5. doi: 10.1186/s12917-018-1732-5 (PMC6318959; doi:10.1186/s12917-018-1732-5)
Supplement: Supplementary file 5 — Figure S3. Heatmap illustrates susceptibility, i.e. susceptible, intermediate, and resistant, of Streptococcus suis, grouped based on serotypes, towards testing antibiotic drugs. The isolated bacteria were clustered into four serotypes, including serotype 2 (n = 59), non-serotype 2 (n = 73), autoagglutinating (n = 91) and non-typable (n = 39). Associations between source of isolation and susceptibility of each antibiotic drug were analyzed using Pearson’s Chi-square dependent test. The asterisk indicates that null hypothesis of the Chi-square test was rejected (P-value < 0.05), suggesting a significant association. (DOCX 70 kb) [file 12917_2018_1732_MOESM5_ESM.docx]

| **Supplementary data** | | | | |  |  |  |  |  |  |  |  |  |  |  |  |  |  |  |  |  |  |  |
| --- | --- | --- | --- | --- | --- | --- | --- | --- | --- | --- | --- | --- | --- | --- | --- | --- | --- | --- | --- | --- | --- | --- | --- |
| **Table S1** | |  |  |  |  |  |  |  |  |  |  |  |  |  |  |  |  |  |  |  |  |  |  |
|  |  |  |  |  |  |  |  |  |  |  |  |  |  |  |  |  |  |  |  |  |  |  |  |
| **No.** | **Strain** |  | **AMR patterns** | | | | | | | | | | | | | | | | | **AMR pattern No.** | **Source** | **Serotype** |  |
| 1 | DP6001 |  | No resistance | | |  |  |  |  |  |  |  |  |  |  |  |  |  |  | 1 | Diseased pig | AA |  |
| 2 | AP161 |  | No resistance | | |  |  |  |  |  |  |  |  |  |  |  |  |  |  | 1 | Asymptomatic pig | NT |  |
| 3 | DP6002 |  | GEN |  |  |  |  |  |  |  |  |  |  |  |  |  |  |  |  | 2 | Diseased pig | NT |  |
| 4 | H001 |  | DOX | TET |  |  |  |  |  |  |  |  |  |  |  |  |  |  |  | 3 | Human patient | 2 |  |
| 5 | DP6003 |  | DOX | TET |  |  |  |  |  |  |  |  |  |  |  |  |  |  |  | 3 | Diseased pig | 34 |  |
| 6 | DP6004 |  | DOX | TET |  |  |  |  |  |  |  |  |  |  |  |  |  |  |  | 3 | Diseased pig | 1 |  |
| 7 | H002 |  | DOX | TET | TIA |  |  |  |  |  |  |  |  |  |  |  |  |  |  | 4 | Human patient | 2 |  |
| 8 | AP011 |  | DOX | TET | TIA | SXT |  |  |  |  |  |  |  |  |  |  |  |  |  | 5 | Asymptomatic pig | 9 |  |
| 9 | AP009 |  | DOX | TET | TIA | LEV |  |  |  |  |  |  |  |  |  |  |  |  |  | 6 | Asymptomatic pig | 22 |  |
| 10 | AP165 |  | DOX | TET | ENR | LEV | SXT |  |  |  |  |  |  |  |  |  |  |  |  | 7 * | Asymptomatic pig | NT |  |
| 11 | H003 |  | DOX | TET | CIP | NOR |  |  |  |  |  |  |  |  |  |  |  |  |  | 8 | Human patient | 2 |  |
| 12 | H004 |  | DOX | TET | CIP | NOR |  |  |  |  |  |  |  |  |  |  |  |  |  | 8 | Human patient | 2 |  |
| 13 | DP6005 |  | DOX | GEN | TIA |  |  |  |  |  |  |  |  |  |  |  |  |  |  | 9 | Diseased pig | NT |  |
| 14 | AP162 |  | DOX | GEN | TET | TIA |  |  |  |  |  |  |  |  |  |  |  |  |  | 10 | Asymptomatic pig | NT |  |
| 15 | H009 |  | DOX | ERY | TET | TIA |  |  |  |  |  |  |  |  |  |  |  |  |  | 11 | Human patient | 2 |  |
| 16 | AP056 |  | CTX | CTF | AZM | CLI | DOX | ERY | TET | TIA | ENR |  |  |  |  |  |  |  |  | 12 * | Asymptomatic pig | 9 |  |
| 17 | AP051 |  | CTX | AZM | CLI | DOX | TET | TIA | LEV |  |  |  |  |  |  |  |  |  |  | 13 * | Asymptomatic pig | 28 |  |
| 18 | AP169 |  | CLI | TIA | LEV | SXT |  |  |  |  |  |  |  |  |  |  |  |  |  | 14 * | Asymptomatic pig | NT |  |
| 19 | AP097 |  | CLI | TET | TIA | SXT |  |  |  |  |  |  |  |  |  |  |  |  |  | 15 | Asymptomatic pig | AA |  |
| 20 | AP090 |  | CLI | DOX | TET | TIA | LEV |  |  |  |  |  |  |  |  |  |  |  |  | 16 | Asymptomatic pig | AA |  |
| 21 | AP019 |  | CLI | DOX | GEN | TET | TIA |  |  |  |  |  |  |  |  |  |  |  |  | 17 | Asymptomatic pig | 28 |  |
| 22 | AP075 |  | CLI | DOX | GEN | TET | TIA |  |  |  |  |  |  |  |  |  |  |  |  | 17 | Asymptomatic pig | AA |  |
| 23 | AP163 |  | CLI | DOX | GEN | TET | TIA |  |  |  |  |  |  |  |  |  |  |  |  | 17 | Asymptomatic pig | NT |  |
| 24 | AP100 |  | CLI | DOX | GEN | TET | TIA | NOR |  |  |  |  |  |  |  |  |  |  |  | 18 | Asymptomatic pig | AA |  |
| 25 | AP010 |  | CLI | DOX | GEN | TET | TIA | SXT |  |  |  |  |  |  |  |  |  |  |  | 19 | Asymptomatic pig | 34 |  |
| 26 | AP101 |  | CLI | DOX | GEN | TET | TIA | CIP | NOR | SXT |  |  |  |  |  |  |  |  |  | 20 | Asymptomatic pig | AA |  |
| 27 | AP044 |  | CLI | DOX | GEN | TET | TIA | NOR | LEV | SXT |  |  |  |  |  |  |  |  |  | 21 * | Asymptomatic pig | 34 |  |
| 28 | DP6016 |  | CFL | VAN | AZM | CLI | DOX | ERY | TET | TIA | ENR | SXT |  |  |  |  |  |  |  | 22 * | Diseased pig | 23 |  |
| 29 | AP126 |  | CFL | PEN | CLI | DOX | TET | TIA | NOR | SXT |  |  |  |  |  |  |  |  |  | 23 * | Asymptomatic pig | AA |  |
| 30 | AP139 |  | CFL | PEN | CLI | DOX | GEN | TET | TIA | LEV |  |  |  |  |  |  |  |  |  | 25 * | Asymptomatic pig | AA |  |
| 31 | AP131 |  | CFL | PEN | AZM | CLI | DOX | GEN | TET | TIA | NOR |  |  |  |  |  |  |  |  | 26 * | Asymptomatic pig | AA |  |
| 32 | AP140 |  | CFL | PEN | AZM | CLI | DOX | ERY | TET | TIA | NOR | LEV |  |  |  |  |  |  |  | 27 * | Asymptomatic pig | AA |  |
| 33 | AP134 |  | CFL | PEN | AZM | CLI | DOX | ERY | TET | TIA | CIP | NOR | SXT |  |  |  |  |  |  | 28 * | Asymptomatic pig | AA |  |
| 34 | AP136 |  | CFL | PEN | AZM | CLI | DOX | ERY | TET | TIA | CIP | ENR | NOR | SXT |  |  |  |  |  | 29 * | Asymptomatic pig | AA |  |
| 35 | AP138 |  | CFL | PEN | AZM | CLI | DOX | GEN | TET | TIA | CIP | ENR | NOR | LEV | SXT |  |  |  |  | 30 * | Asymptomatic pig | AA |  |

| **Supplementary data** | | | | |  |  |  |  |  |  |  |  |  |  |  |  |  |  |  |  |  |  |  |
| --- | --- | --- | --- | --- | --- | --- | --- | --- | --- | --- | --- | --- | --- | --- | --- | --- | --- | --- | --- | --- | --- | --- | --- |
| **Table S1 (continued)** | | | | |  |  |  |  |  |  |  |  |  |  |  |  |  |  |  |  |  |  |  |
|  |  |  |  |  |  |  |  |  |  |  |  |  |  |  |  |  |  |  |  |  |  |  |  |
| **No.** | **Strain** |  | **AMR patterns** | | | | | | | | | | | | | | | | | **AMR pattern No.** | **Source** | **Serotype** |  |
| 36 | AP058 |  | CFL | PEN | AZM | CLI | DOX | ERY | GEN | TET | TIA | SXT |  |  |  |  |  |  |  | 31 * | Asymptomatic pig | 29 |  |
| 37 | AP183 |  | CFL | PEN | AZM | CLI | DOX | ERY | GEN | TET | TIA | SXT |  |  |  |  |  |  |  | 31 * | Asymptomatic pig | NT |  |
| 38 | AP059 |  | CFL | PEN | AZM | CLI | DOX | ERY | GEN | TET | TIA | CIP | ENR | NOR |  |  |  |  |  | 32 * | Asymptomatic pig | 22 |  |
| 39 | AP137 |  | CFL | PEN | AZM | CLI | DOX | ERY | GEN | TET | TIA | CIP | ENR | NOR | SXT |  |  |  |  | 33 * | Asymptomatic pig | AA |  |
| 40 | AP184 |  | CFL | PEN | AZM | CLI | DOX | ERY | GEN | TET | TIA | CIP | ENR | NOR | LEV | SXT |  |  |  | 34 * | Asymptomatic pig | NT |  |
| 41 | AP132 |  | CFL | PEN | AZM | CHL | CLI | DOX | ERY | TET | TIA | SXT |  |  |  |  |  |  |  | 35 * | Asymptomatic pig | AA |  |
| 42 | AP135 |  | CFL | PEN | AZM | CHL | CLI | DOX | ERY | GEN | TET | TIA | SXT |  |  |  |  |  |  | 36 * | Asymptomatic pig | AA |  |
| 43 | AP124 |  | CFL | GEN |  |  |  |  |  |  |  |  |  |  |  |  |  |  |  | 37 | Asymptomatic pig | AA |  |
| 44 | AP076 |  | CFL | GEN | CIP | NOR |  |  |  |  |  |  |  |  |  |  |  |  |  | 38 * | Asymptomatic pig | AA |  |
| 45 | AP181 |  | CFL | CTX | VAN | CLI | DOX | TET | TIA | CIP | ENR | SXT |  |  |  |  |  |  |  | 39 * | Asymptomatic pig | NT |  |
| 46 | DP6008 |  | CFL | CTX | CTF | VAN | CLI | GEN | TIA |  |  |  |  |  |  |  |  |  |  | 40 | Diseased pig | NT |  |
| 47 | DP1523 |  | CFL | CTX | CTF | PEN | AZM | CLI | DOX | ERY | TET | TIA | SXT |  |  |  |  |  |  | 41 * | Diseased pig | 2 |  |
| 48 | AP055 |  | CFL | CTX | CTF | AZM | CLI | DOX | ERY | TET | TIA |  |  |  |  |  |  |  |  | 42 | Asymptomatic pig | 30 |  |
| 49 | AP182 |  | CFL | CTX | CTF | AZM | CLI | DOX | ERY | TET | TIA | ENR |  |  |  |  |  |  |  | 43 * | Asymptomatic pig | NT |  |
| 50 | DP6011 |  | CFL | CTX | CLI | DOX | GEN | TET | TIA | CIP | ENR | NOR |  |  |  |  |  |  |  | 44 * | Diseased pig | NT |  |
| 51 | AP052 |  | CFL | CTX | AZM | CLI | DOX | ERY | GEN | TET | TIA | CIP | ENR | NOR | LEV | SXT |  |  |  | 45 * | Asymptomatic pig | 25 |  |
| 52 | DP6021 |  | CFL | CTX | AZM | CHL | CLI | DOX | ERY | FFC | TET | TIA | CIP | ENR | NOR | SXT |  |  |  | 46 * | Diseased pig | AA |  |
| 53 | AP091 |  | CFL | CTF | CLI | DOX | ERY | GEN | TIA |  |  |  |  |  |  |  |  |  |  | 47 | Asymptomatic pig | AA |  |
| 54 | AP123 |  | CFL | CTF | CLI | DOX | ERY | FFC | TET | TIA | ENR | NOR | SXT |  |  |  |  |  |  | 48 * | Asymptomatic pig | AA |  |
| 55 | DP6009 |  | CFL | CLI | DOX | GEN | TET | TIA | CIP | ENR | NOR |  |  |  |  |  |  |  |  | 49 * | Diseased pig | NT |  |
| 56 | AP180 |  | CFL | CLI | DOX | GEN | TET | TIA | CIP | ENR | NOR | LEV | SXT |  |  |  |  |  |  | 50 * | Asymptomatic pig | NT |  |
| 57 | AP031 |  | CFL | AZM | CLI | ERY | TIA | LEV | SXT |  |  |  |  |  |  |  |  |  |  | 51 * | Asymptomatic pig | 19 |  |
| 58 | AP099 |  | CFL | AZM | CLI | ERY | TIA | LEV | SXT |  |  |  |  |  |  |  |  |  |  | 51 * | Asymptomatic pig | AA |  |
| 59 | AP037 |  | CFL | AZM | CLI | ERY | TIA | NOR | LEV | SXT |  |  |  |  |  |  |  |  |  | 52 * | Asymptomatic pig | 19 |  |
| 60 | AP053 |  | CFL | AZM | CLI | ERY | TET | TIA |  |  |  |  |  |  |  |  |  |  |  | 53 * | Asymptomatic pig | 22 |  |
| 61 | AP120 |  | CFL | AZM | CLI | ERY | FFC | GEN | TET | TIA | CIP | ENR | NOR | SXT |  |  |  |  |  | 54 * | Asymptomatic pig | AA |  |
| 62 | AP128 |  | CFL | AZM | CLI | DOX | ERY | GEN | TET | TIA | SXT |  |  |  |  |  |  |  |  | 55 * | Asymptomatic pig | AA |  |
| 63 | AP054 |  | CFL | AZM | CLI | DOX | ERY | GEN | TET | TIA | CIP | ENR | NOR |  |  |  |  |  |  | 56 | Asymptomatic pig | 22 |  |
| 64 | AP117 |  | CFL | AZM | CLI | DOX | ERY | GEN | TET | TIA | CIP | ENR | NOR | SXT |  |  |  |  |  | 57 * | Asymptomatic pig | AA |  |
| 65 | AP121 |  | CFL | AZM | CLI | DOX | ERY | GEN | TET | TIA | CIP | ENR | NOR | SXT |  |  |  |  |  | 57 * | Asymptomatic pig | AA |  |
| 66 | AP050 |  | CFL | AZM | CLI | DOX | ERY | GEN | TET | TIA | CIP | ENR | NOR | LEV |  |  |  |  |  | 58 * | Asymptomatic pig | 21 |  |
| 67 | AP122 |  | CFL | AZM | CLI | DOX | ERY | FFC | GEN | TET | TIA | CIP | ENR | NOR | SXT |  |  |  |  | 59 * | Asymptomatic pig | AA |  |
| 68 | AP179 |  | CFL | AZM | CLI | DOX | ERY | TET | TIA | SXT |  |  |  |  |  |  |  |  |  | 60 * | Asymptomatic pig | NT |  |
| 69 | AP125 |  | CFL | AZM | CLI | DOX | ERY | TET | TIA | NOR | SXT |  |  |  |  |  |  |  |  | 61 * | Asymptomatic pig | AA |  |
| 70 | AP168 |  | CFL | AZM | CLI | DOX | ERY | TET | TIA | NOR | LEV | SXT |  |  |  |  |  |  |  | 62 * | Asymptomatic pig | NT |  |

| **Supplementary data** | | | | |  |  |  |  |  |  |  |  |  |  |  |  |  |  |  |  |  |  |  |
| --- | --- | --- | --- | --- | --- | --- | --- | --- | --- | --- | --- | --- | --- | --- | --- | --- | --- | --- | --- | --- | --- | --- | --- |
| **Table S1 (continued)** | | | | |  |  |  |  |  |  |  |  |  |  |  |  |  |  |  |  |  |  |  |
|  |  |  |  |  |  |  |  |  |  |  |  |  |  |  |  |  |  |  |  |  |  |  |  |
| **No.** | **Strain** |  | **AMR patterns** | | | | | | | | | | | | | | | | | **AMR pattern No.** | **Source** | **Serotype** |  |
| 71 | DP6017 |  | CFL | AZM | CLI | DOX | ERY | TET | TIA | CIP | NOR |  |  |  |  |  |  |  |  | 63 * | Diseased pig | NT |  |
| 72 | AP046 |  | CFL | AZM | CLI | DOX | ERY | TET | TIA | CIP | ENR | NOR | SXT |  |  |  |  |  |  | 64 * | Asymptomatic pig | 3 |  |
| 73 | AP096 |  | CFL | AZM | CLI | DOX | ERY | TET | TIA | CIP | ENR | NOR | LEV | SXT |  |  |  |  |  | 65 * | Asymptomatic pig | AA |  |
| 74 | AP178 |  | CFL | AZM | CLI | DOX | ERY | TET | TIA | CIP | ENR | NOR | LEV | SXT |  |  |  |  |  | 65 * | Asymptomatic pig | NT |  |
| 75 | DP6006 |  | AZM | CLI | ERY | TIA | SXT |  |  |  |  |  |  |  |  |  |  |  |  | 66 | Diseased pig | NT |  |
| 76 | AP008 |  | AZM | CLI | ERY | TIA | SXT |  |  |  |  |  |  |  |  |  |  |  |  | 66 | Asymptomatic pig | 22 |  |
| 77 | AP073 |  | AZM | CLI | ERY | TIA | SXT |  |  |  |  |  |  |  |  |  |  |  |  | 66 | Asymptomatic pig | AA |  |
| 78 | AP074 |  | AZM | CLI | ERY | TIA | SXT |  |  |  |  |  |  |  |  |  |  |  |  | 66 | Asymptomatic pig | AA |  |
| 79 | AP020 |  | AZM | CLI | ERY | TIA | NOR | LEV | SXT |  |  |  |  |  |  |  |  |  |  | 67 * | Asymptomatic pig | 22 |  |
| 80 | AP013 |  | AZM | CLI | ERY | TIA | NOR |  |  |  |  |  |  |  |  |  |  |  |  | 68 | Asymptomatic pig | 22 |  |
| 81 | AP176 |  | AZM | CLI | ERY | TIA | ENR |  |  |  |  |  |  |  |  |  |  |  |  | 69 | Asymptomatic pig | NT |  |
| 82 | AP079 |  | AZM | CLI | ERY | TET | TIA | LEV | SXT |  |  |  |  |  |  |  |  |  |  | 70 * | Asymptomatic pig | AA |  |
| 83 | AP045 |  | AZM | CLI | ERY | TET | TIA | CIP | NOR | LEV | SXT |  |  |  |  |  |  |  |  | 71 * | Asymptomatic pig | 16 |  |
| 84 | AP164 |  | AZM | CLI | ERY | SXT |  |  |  |  |  |  |  |  |  |  |  |  |  | 72 | Asymptomatic pig | NT |  |
| 85 | AP043 |  | AZM | CLI | ERY | GEN | TET | TIA | SXT |  |  |  |  |  |  |  |  |  |  | 73 | Asymptomatic pig | 12 |  |
| 86 | H005 |  | AZM | CLI | DOX | TET |  |  |  |  |  |  |  |  |  |  |  |  |  | 74 | Human patient | 2 |  |
| 87 | H006 |  | AZM | CLI | DOX | TET |  |  |  |  |  |  |  |  |  |  |  |  |  | 74 | Human patient | 2 |  |
| 88 | H007 |  | AZM | CLI | DOX | TET |  |  |  |  |  |  |  |  |  |  |  |  |  | 74 | Human patient | 2 |  |
| 89 | H008 |  | AZM | CLI | DOX | TET |  |  |  |  |  |  |  |  |  |  |  |  |  | 74 | Human patient | 2 |  |
| 90 | DP6012 |  | AZM | CLI | DOX | TET | NOR |  |  |  |  |  |  |  |  |  |  |  |  | 75 | Diseased pig | 2 |  |
| 91 | AP004 |  | AZM | CLI | DOX | ERY | TET | TIA | NOR |  |  |  |  |  |  |  |  |  |  | 76 | Asymptomatic pig | 2 |  |
| 92 | AP035 |  | AZM | CLI | DOX | ERY | TET | TIA | NOR |  |  |  |  |  |  |  |  |  |  | 76 | Asymptomatic pig | 30 |  |
| 93 | AP172 |  | AZM | CLI | DOX | ERY | TET | TIA | NOR |  |  |  |  |  |  |  |  |  |  | 76 | Asymptomatic pig | NT |  |
| 94 | AP175 |  | AZM | CLI | DOX | ERY | TET | TIA | NOR |  |  |  |  |  |  |  |  |  |  | 76 | Asymptomatic pig | NT |  |
| 95 | DP6014 |  | AZM | CLI | DOX | ERY | TET | TIA | SXT |  |  |  |  |  |  |  |  |  |  | 77 | Diseased pig | 14 |  |
| 96 | AP026 |  | AZM | CLI | DOX | ERY | TET | TIA | SXT |  |  |  |  |  |  |  |  |  |  | 77 | Asymptomatic pig | 5 |  |
| 97 | AP027 |  | AZM | CLI | DOX | ERY | TET | TIA | SXT |  |  |  |  |  |  |  |  |  |  | 77 | Asymptomatic pig | 27 |  |
| 98 | AP028 |  | AZM | CLI | DOX | ERY | TET | TIA | SXT |  |  |  |  |  |  |  |  |  |  | 77 | Asymptomatic pig | 30 |  |
| 99 | AP039 |  | AZM | CLI | DOX | ERY | TET | TIA | SXT |  |  |  |  |  |  |  |  |  |  | 77 | Asymptomatic pig | 5 |  |
| 100 | AP041 |  | AZM | CLI | DOX | ERY | TET | TIA | SXT |  |  |  |  |  |  |  |  |  |  | 77 | Asymptomatic pig | 9 |  |
| 101 | AP171 |  | AZM | CLI | DOX | ERY | TET | TIA | SXT |  |  |  |  |  |  |  |  |  |  | 77 | Asymptomatic pig | NT |  |
| 102 | H010 |  | AZM | CLI | DOX | ERY | TET |  |  |  |  |  |  |  |  |  |  |  |  | 78 | Human patient | 2 |  |
| 103 | H011 |  | AZM | CLI | DOX | ERY | TET |  |  |  |  |  |  |  |  |  |  |  |  | 78 | Human patient | 2 |  |
| 104 | H012 |  | AZM | CLI | DOX | ERY | TET |  |  |  |  |  |  |  |  |  |  |  |  | 78 | Human patient | 2 |  |
| 105 | H013 |  | AZM | CLI | DOX | ERY | TET |  |  |  |  |  |  |  |  |  |  |  |  | 78 | Human patient | 2 |  |

| **Supplementary data** | | | | |  |  |  |  |  |  |  |  |  |  |  |  |  |  |  |  |  |  |  |
| --- | --- | --- | --- | --- | --- | --- | --- | --- | --- | --- | --- | --- | --- | --- | --- | --- | --- | --- | --- | --- | --- | --- | --- |
| **Table S1 (continued)** | | | | |  |  |  |  |  |  |  |  |  |  |  |  |  |  |  |  |  |  |  |
|  |  |  |  |  |  |  |  |  |  |  |  |  |  |  |  |  |  |  |  |  |  |  |  |
| **No.** | **Strain** |  | **AMR patterns** | | | | | | | | | | | | | | | | | **AMR pattern No.** | **Source** | **Serotype** |  |
| 106 | H014 |  | AZM | CLI | DOX | ERY | TET |  |  |  |  |  |  |  |  |  |  |  |  | 78 | Human patient | 2 |  |
| 107 | H015 |  | AZM | CLI | DOX | ERY | TET |  |  |  |  |  |  |  |  |  |  |  |  | 78 | Human patient | 2 |  |
| 108 | H016 |  | AZM | CLI | DOX | ERY | TET |  |  |  |  |  |  |  |  |  |  |  |  | 78 | Human patient | 2 |  |
| 109 | H017 |  | AZM | CLI | DOX | ERY | TET |  |  |  |  |  |  |  |  |  |  |  |  | 78 | Human patient | 2 |  |
| 110 | H019 |  | AZM | CLI | DOX | ERY | TET |  |  |  |  |  |  |  |  |  |  |  |  | 78 | Human patient | 2 |  |
| 111 | H020 |  | AZM | CLI | DOX | ERY | TET |  |  |  |  |  |  |  |  |  |  |  |  | 78 | Human patient | 2 |  |
| 112 | H021 |  | AZM | CLI | DOX | ERY | TET |  |  |  |  |  |  |  |  |  |  |  |  | 78 | Human patient | 2 |  |
| 113 | H022 |  | AZM | CLI | DOX | ERY | TET |  |  |  |  |  |  |  |  |  |  |  |  | 78 | Human patient | 2 |  |
| 114 | H023 |  | AZM | CLI | DOX | ERY | TET |  |  |  |  |  |  |  |  |  |  |  |  | 78 | Human patient | 2 |  |
| 115 | AP001 |  | AZM | CLI | DOX | ERY | TET |  |  |  |  |  |  |  |  |  |  |  |  | 78 | Asymptomatic pig | 2 |  |
| 116 | AP003 |  | AZM | CLI | DOX | ERY | TET |  |  |  |  |  |  |  |  |  |  |  |  | 78 | Asymptomatic pig | 2 |  |
| 117 | AP012 |  | AZM | CLI | DOX | ERY | TET |  |  |  |  |  |  |  |  |  |  |  |  | 78 | Asymptomatic pig | 3 |  |
| 118 | AP014 |  | AZM | CLI | DOX | ERY | TET |  |  |  |  |  |  |  |  |  |  |  |  | 78 | Asymptomatic pig | 3 |  |
| 119 | AP015 |  | AZM | CLI | DOX | ERY | TET |  |  |  |  |  |  |  |  |  |  |  |  | 78 | Asymptomatic pig | 3 |  |
| 120 | AP016 |  | AZM | CLI | DOX | ERY | TET |  |  |  |  |  |  |  |  |  |  |  |  | 78 | Asymptomatic pig | 3 |  |
| 121 | AP017 |  | AZM | CLI | DOX | ERY | TET |  |  |  |  |  |  |  |  |  |  |  |  | 78 | Asymptomatic pig | 7 |  |
| 122 | AP078 |  | AZM | CLI | DOX | ERY | TET |  |  |  |  |  |  |  |  |  |  |  |  | 78 | Asymptomatic pig | AA |  |
| 123 | AP166 |  | AZM | CLI | DOX | ERY | TET |  |  |  |  |  |  |  |  |  |  |  |  | 78 | Asymptomatic pig | NT |  |
| 124 | H018 |  | AZM | CLI | DOX | ERY | TET | TIA |  |  |  |  |  |  |  |  |  |  |  | 79 | Human patient | 2 |  |
| 125 | H027 |  | AZM | CLI | DOX | ERY | TET | TIA |  |  |  |  |  |  |  |  |  |  |  | 79 | Human patient | 2 |  |
| 126 | DP1501 |  | AZM | CLI | DOX | ERY | TET | TIA |  |  |  |  |  |  |  |  |  |  |  | 79 | Diseased pig | 2 |  |
| 127 | DP1503 |  | AZM | CLI | DOX | ERY | TET | TIA |  |  |  |  |  |  |  |  |  |  |  | 79 | Diseased pig | 2 |  |
| 128 | DP1522 |  | AZM | CLI | DOX | ERY | TET | TIA |  |  |  |  |  |  |  |  |  |  |  | 79 | Diseased pig | 2 |  |
| 129 | AP002 |  | AZM | CLI | DOX | ERY | TET | TIA |  |  |  |  |  |  |  |  |  |  |  | 79 | Asymptomatic pig | 2 |  |
| 130 | AP005 |  | AZM | CLI | DOX | ERY | TET | TIA |  |  |  |  |  |  |  |  |  |  |  | 79 | Asymptomatic pig | 2 |  |
| 131 | AP018 |  | AZM | CLI | DOX | ERY | TET | TIA |  |  |  |  |  |  |  |  |  |  |  | 79 | Asymptomatic pig | 9 |  |
| 132 | AP077 |  | AZM | CLI | DOX | ERY | TET | TIA |  |  |  |  |  |  |  |  |  |  |  | 79 | Asymptomatic pig | AA |  |
| 133 | AP080 |  | AZM | CLI | DOX | ERY | TET | TIA |  |  |  |  |  |  |  |  |  |  |  | 79 | Asymptomatic pig | AA |  |
| 134 | AP113 |  | AZM | CLI | DOX | ERY | TET | TIA |  |  |  |  |  |  |  |  |  |  |  | 79 | Asymptomatic pig | AA |  |
| 135 | AP081 |  | AZM | CLI | DOX | ERY | TET | TIA | SXT |  |  |  |  |  |  |  |  |  |  | 80 | Asymptomatic pig | AA |  |
| 136 | AP082 |  | AZM | CLI | DOX | ERY | TET | TIA | SXT |  |  |  |  |  |  |  |  |  |  | 80 | Asymptomatic pig | AA |  |
| 137 | AP085 |  | AZM | CLI | DOX | ERY | TET | TIA | SXT |  |  |  |  |  |  |  |  |  |  | 80 | Asymptomatic pig | AA |  |
| 138 | AP098 |  | AZM | CLI | DOX | ERY | TET | TIA | SXT |  |  |  |  |  |  |  |  |  |  | 80 | Asymptomatic pig | AA |  |
| 139 | AP102 |  | AZM | CLI | DOX | ERY | TET | TIA | SXT |  |  |  |  |  |  |  |  |  |  | 80 | Asymptomatic pig | AA |  |
| 140 | AP103 |  | AZM | CLI | DOX | ERY | TET | TIA | SXT |  |  |  |  |  |  |  |  |  |  | 80 | Asymptomatic pig | AA |  |
| 141 | AP104 |  | AZM | CLI | DOX | ERY | TET | TIA | SXT |  |  |  |  |  |  |  |  |  |  | 80 | Asymptomatic pig | AA |  |
| 142 | AP107 |  | AZM | CLI | DOX | ERY | TET | TIA | SXT |  |  |  |  |  |  |  |  |  |  | 80 | Asymptomatic pig | AA |  |

| **Supplementary data** | | | | |  |  |  |  |  |  |  |  |  |  |  |  |  |  |  |  |  |  |  |
| --- | --- | --- | --- | --- | --- | --- | --- | --- | --- | --- | --- | --- | --- | --- | --- | --- | --- | --- | --- | --- | --- | --- | --- |
| **Table S1 (continued)** | | | | |  |  |  |  |  |  |  |  |  |  |  |  |  |  |  |  |  |  |  |
|  |  |  |  |  |  |  |  |  |  |  |  |  |  |  |  |  |  |  |  |  |  |  |  |
| **No.** | **Strain** |  | **AMR patterns** | | | | | | | | | | | | | | | | | **AMR pattern No.** | **Source** | **Serotype** |  |
| 143 | AP110 |  | AZM | CLI | DOX | ERY | TET | TIA | SXT |  |  |  |  |  |  |  |  |  |  | 80 | Asymptomatic pig | AA |  |
| 144 | H025 |  | AZM | CLI | DOX | ERY | TET | NOR |  |  |  |  |  |  |  |  |  |  |  | 81 | Human patient | 2 |  |
| 145 | AP022 |  | AZM | CLI | DOX | ERY | TET | NOR |  |  |  |  |  |  |  |  |  |  |  | 81 | Asymptomatic pig | 7 |  |
| 146 | AP006 |  | AZM | CLI | DOX | ERY | TET | CIP | NOR |  |  |  |  |  |  |  |  |  |  | 82 | Asymptomatic pig | 2 |  |
| 147 | AP007 |  | AZM | CLI | DOX | ERY | TET | CIP | NOR |  |  |  |  |  |  |  |  |  |  | 82 | Asymptomatic pig | 2 |  |
| 148 | DP1502 |  | AZM | CLI | DOX | ERY | TET | SXT |  |  |  |  |  |  |  |  |  |  |  | 83 | Diseased pig | 2 |  |
| 149 | DP6007 |  | AZM | CLI | DOX | ERY | TET | TIA | LEV | SXT |  |  |  |  |  |  |  |  |  | 84 * | Diseased pig | NT |  |
| 150 | AP042 |  | AZM | CLI | DOX | ERY | TET | TIA | LEV | SXT |  |  |  |  |  |  |  |  |  | 84 * | Asymptomatic pig | 9 |  |
| 151 | AP083 |  | AZM | CLI | DOX | ERY | TET | TIA | LEV | SXT |  |  |  |  |  |  |  |  |  | 84 * | Asymptomatic pig | AA |  |
| 152 | AP084 |  | AZM | CLI | DOX | ERY | TET | TIA | LEV | SXT |  |  |  |  |  |  |  |  |  | 84 * | Asymptomatic pig | AA |  |
| 153 | AP086 |  | AZM | CLI | DOX | ERY | TET | TIA | LEV | SXT |  |  |  |  |  |  |  |  |  | 84 * | Asymptomatic pig | AA |  |
| 154 | AP109 |  | AZM | CLI | DOX | ERY | TET | TIA | LEV | SXT |  |  |  |  |  |  |  |  |  | 84 * | Asymptomatic pig | AA |  |
| 155 | AP092 |  | AZM | CLI | DOX | ERY | TET | TIA | LEV | SXT |  |  |  |  |  |  |  |  |  | 84 * | Asymptomatic pig | AA |  |
| 156 | AP034 |  | AZM | CLI | DOX | ERY | TET | TIA | NOR | SXT |  |  |  |  |  |  |  |  |  | 85 * | Asymptomatic pig | 16 |  |
| 157 | AP040 |  | AZM | CLI | DOX | ERY | TET | TIA | NOR | SXT |  |  |  |  |  |  |  |  |  | 85 * | Asymptomatic pig | 5 |  |
| 158 | AP087 |  | AZM | CLI | DOX | ERY | TET | TIA | NOR | SXT |  |  |  |  |  |  |  |  |  | 85 * | Asymptomatic pig | AA |  |
| 159 | AP105 |  | AZM | CLI | DOX | ERY | TET | TIA | NOR | SXT |  |  |  |  |  |  |  |  |  | 85 * | Asymptomatic pig | AA |  |
| 160 | AP170 |  | AZM | CLI | DOX | ERY | TET | TIA | NOR | SXT |  |  |  |  |  |  |  |  |  | 85 * | Asymptomatic pig | NT |  |
| 161 | AP174 |  | AZM | CLI | DOX | ERY | TET | TIA | NOR | SXT |  |  |  |  |  |  |  |  |  | 85 * | Asymptomatic pig | NT |  |
| 162 | AP177 |  | AZM | CLI | DOX | ERY | TET | TIA | NOR | SXT |  |  |  |  |  |  |  |  |  | 85 * | Asymptomatic pig | NT |  |
| 163 | AP152 |  | AZM | CLI | DOX | ERY | TET | TIA | CIP | NOR | SXT |  |  |  |  |  |  |  |  | 86 * | Asymptomatic pig | AA |  |
| 164 | AP033 |  | AZM | CLI | DOX | ERY | TET | TIA | NOR | LEV | SXT |  |  |  |  |  |  |  |  | 87 * | Asymptomatic pig | 22 |  |
| 165 | DP6010 |  | AZM | CLI | DOX | ERY | TET | TIA | CIP | ENR | NOR | SXT |  |  |  |  |  |  |  | 88 * | Diseased pig | NT |  |
| 166 | AP094 |  | AZM | CLI | DOX | ERY | TET | TIA | CIP | ENR | NOR | SXT |  |  |  |  |  |  |  | 88 * | Asymptomatic pig | AA |  |
| 167 | AP127 |  | AZM | CLI | DOX | ERY | TET | TIA | CIP | ENR | NOR | SXT |  |  |  |  |  |  |  | 88 * | Asymptomatic pig | AA |  |
| 168 | AP118 |  | AZM | CLI | DOX | ERY | TET | TIA | CIP | ENR | NOR | LEV |  |  |  |  |  |  |  | 89 | Asymptomatic pig | AA |  |
| 169 | AP049 |  | AZM | CLI | DOX | ERY | TET | TIA | CIP | ENR | NOR | LEV | SXT |  |  |  |  |  |  | 90 * | Asymptomatic pig | 22 |  |
| 170 | AP167 |  | AZM | CLI | DOX | ERY | TET | TIA | CIP | ENR | NOR | LEV | SXT |  |  |  |  |  |  | 90 * | Asymptomatic pig | NT |  |
| 171 | DP6013 |  | AZM | CLI | DOX | ERY | GEN | TET | TIA |  |  |  |  |  |  |  |  |  |  | 91 | Diseased pig | 2 |  |
| 172 | DP1507 |  | AZM | CLI | DOX | ERY | GEN | TET | TIA |  |  |  |  |  |  |  |  |  |  | 91 | Diseased pig | 2 |  |
| 173 | DP1509 |  | AZM | CLI | DOX | ERY | GEN | TET | TIA |  |  |  |  |  |  |  |  |  |  | 91 | Diseased pig | 2 |  |
| 174 | AP095 |  | AZM | CLI | DOX | ERY | GEN | TET | TIA |  |  |  |  |  |  |  |  |  |  | 91 | Asymptomatic pig | AA |  |
| 175 | H024 |  | AZM | CLI | DOX | ERY | GEN | TET |  |  |  |  |  |  |  |  |  |  |  | 92 | Human patient | 2 |  |
| 176 | H026 |  | AZM | CLI | DOX | ERY | GEN | TET |  |  |  |  |  |  |  |  |  |  |  | 92 | Human patient | 2 |  |
| 177 | DP1508 |  | AZM | CLI | DOX | ERY | GEN | TET | SXT |  |  |  |  |  |  |  |  |  |  | 93 | Diseased pig | 2 |  |
| 178 | AP023 |  | AZM | CLI | DOX | ERY | GEN | TET | TIA | SXT |  |  |  |  |  |  |  |  |  | 94 | Asymptomatic pig | 12 |  |
| 179 | AP036 |  | AZM | CLI | DOX | ERY | GEN | TET | TIA | SXT |  |  |  |  |  |  |  |  |  | 94 | Asymptomatic pig | 15 |  |

| **Supplementary data** | | | | |  |  |  |  |  |  |  |  |  |  |  |  |  |  |  |  |  |  |  |
| --- | --- | --- | --- | --- | --- | --- | --- | --- | --- | --- | --- | --- | --- | --- | --- | --- | --- | --- | --- | --- | --- | --- | --- |
| **Table S1 (continued)** | | | | |  |  |  |  |  |  |  |  |  |  |  |  |  |  |  |  |  |  |  |
|  |  |  |  |  |  |  |  |  |  |  |  |  |  |  |  |  |  |  |  |  |  |  |  |
| **No.** | **Strain** |  | **AMR patterns** | | | | | | | | | | | | | | | | | **AMR pattern No.** | **Source** | **Serotype** |  |
| 180 | AP173 |  | AZM | CLI | DOX | ERY | GEN | TET | TIA | SXT |  |  |  |  |  |  |  |  |  | 94 | Asymptomatic pig | NT |  |
| 181 | DP1511 |  | AZM | CLI | DOX | ERY | GEN | TET | NOR | SXT |  |  |  |  |  |  |  |  |  | 95 * | Diseased pig | 2 |  |
| 182 | DP1510 |  | AZM | CLI | DOX | ERY | GEN | TET | TIA | NOR |  |  |  |  |  |  |  |  |  | 96 | Diseased pig | 2 |  |
| 183 | DP1512 |  | AZM | CLI | DOX | ERY | GEN | TET | TIA | NOR |  |  |  |  |  |  |  |  |  | 96 | Diseased pig | 2 |  |
| 184 | DP6015 |  | AZM | CLI | DOX | ERY | GEN | TET | TIA | NOR | SXT |  |  |  |  |  |  |  |  | 97 * | Diseased pig | 16 |  |
| 185 | DP1515 |  | AZM | CLI | DOX | ERY | GEN | TET | TIA | NOR | SXT |  |  |  |  |  |  |  |  | 97 * | Diseased pig | 2 |  |
| 186 | DP1516 |  | AZM | CLI | DOX | ERY | GEN | TET | TIA | NOR | SXT |  |  |  |  |  |  |  |  | 97 * | Diseased pig | 2 |  |
| 187 | DP1517 |  | AZM | CLI | DOX | ERY | GEN | TET | TIA | NOR | SXT |  |  |  |  |  |  |  |  | 97 * | Diseased pig | 2 |  |
| 188 | DP1518 |  | AZM | CLI | DOX | ERY | GEN | TET | TIA | NOR | SXT |  |  |  |  |  |  |  |  | 97 * | Diseased pig | 2 |  |
| 189 | DP1519 |  | AZM | CLI | DOX | ERY | GEN | TET | TIA | NOR | SXT |  |  |  |  |  |  |  |  | 97 * | Diseased pig | 2 |  |
| 190 | AP021 |  | AZM | CLI | DOX | ERY | GEN | TET | TIA | NOR | SXT |  |  |  |  |  |  |  |  | 97 * | Asymptomatic pig | 1 |  |
| 191 | AP088 |  | AZM | CLI | DOX | ERY | GEN | TET | TIA | NOR | SXT |  |  |  |  |  |  |  |  | 97 * | Asymptomatic pig | AA |  |
| 192 | AP106 |  | AZM | CLI | DOX | ERY | GEN | TET | TIA | NOR | SXT |  |  |  |  |  |  |  |  | 97 * | Asymptomatic pig | AA |  |
| 193 | AP108 |  | AZM | CLI | DOX | ERY | GEN | TET | TIA | NOR | SXT |  |  |  |  |  |  |  |  | 97 * | Asymptomatic pig | AA |  |
| 194 | AP111 |  | AZM | CLI | DOX | ERY | GEN | TET | TIA | NOR | SXT |  |  |  |  |  |  |  |  | 97 * | Asymptomatic pig | AA |  |
| 195 | AP112 |  | AZM | CLI | DOX | ERY | GEN | TET | TIA | NOR | SXT |  |  |  |  |  |  |  |  | 97 * | Asymptomatic pig | AA |  |
| 196 | AP093 |  | AZM | CLI | DOX | ERY | GEN | TET | CIP | NOR | SXT |  |  |  |  |  |  |  |  | 98 * | Asymptomatic pig | AA |  |
| 197 | AP114 |  | AZM | CLI | DOX | ERY | GEN | TET | TIA | NOR | LEV | SXT |  |  |  |  |  |  |  | 99 * | Asymptomatic pig | AA |  |
| 198 | AP115 |  | AZM | CLI | DOX | ERY | GEN | TET | TIA | NOR | LEV | SXT |  |  |  |  |  |  |  | 99 * | Asymptomatic pig | AA |  |
| 199 | AP116 |  | AZM | CLI | DOX | ERY | GEN | TET | TIA | NOR | LEV | SXT |  |  |  |  |  |  |  | 99 * | Asymptomatic pig | AA |  |
| 200 | AP038 |  | AZM | CLI | DOX | ERY | GEN | TET | TIA | LEV |  |  |  |  |  |  |  |  |  | 100 | Asymptomatic pig | 24 |  |
| 201 | AP030 |  | AZM | CLI | DOX | ERY | GEN | TET | TIA | LEV | SXT |  |  |  |  |  |  |  |  | 101 * | Asymptomatic pig | 30 |  |
| 202 | AP032 |  | AZM | CLI | DOX | ERY | GEN | TET | TIA | LEV | SXT |  |  |  |  |  |  |  |  | 101 * | Asymptomatic pig | 30 |  |
| 203 | DP1520 |  | AZM | CLI | DOX | ERY | GEN | TET | TIA | CIP | NOR | SXT |  |  |  |  |  |  |  | 102 * | Diseased pig | 2 |  |
| 204 | AP029 |  | AZM | CLI | DOX | ERY | GEN | TET | TIA | CIP | NOR | SXT |  |  |  |  |  |  |  | 102 * | Asymptomatic pig | 34 |  |
| 205 | AP089 |  | AZM | CLI | DOX | ERY | GEN | TET | TIA | CIP | ENR | NOR |  |  |  |  |  |  |  | 103 | Asymptomatic pig | AA |  |
| 206 | AP024 |  | AZM | CLI | DOX | ERY | GEN | TET | TIA | CIP | ENR | NOR | SXT |  |  |  |  |  |  | 104 * | Asymptomatic pig | 16 |  |
| 207 | AP025 |  | AZM | CLI | DOX | ERY | GEN | TET | TIA | CIP | ENR | NOR | SXT |  |  |  |  |  |  | 104 * | Asymptomatic pig | 16 |  |
| 208 | AP047 |  | AZM | CLI | DOX | ERY | GEN | TET | TIA | CIP | ENR | NOR | SXT |  |  |  |  |  |  | 104 * | Asymptomatic pig | 22 |  |
| 209 | AP048 |  | AZM | CLI | DOX | ERY | GEN | TET | TIA | CIP | ENR | NOR | SXT |  |  |  |  |  |  | 104 * | Asymptomatic pig | 16 |  |
| 210 | AP119 |  | AZM | CLI | DOX | ERY | GEN | TET | TIA | CIP | ENR | NOR | LEV | SXT |  |  |  |  |  | 105 * | Asymptomatic pig | AA |  |
| 211 | DP1504 |  | AZM | CLI | DOX | ERY | FFC | TET | TIA |  |  |  |  |  |  |  |  |  |  | 106 | Diseased pig | 2 |  |
| 212 | DP1505 |  | AZM | CLI | DOX | ERY | FFC | TET | TIA |  |  |  |  |  |  |  |  |  |  | 106 | Diseased pig | 2 |  |
| 213 | DP1506 |  | AZM | CLI | DOX | ERY | FFC | TET | TIA |  |  |  |  |  |  |  |  |  |  | 106 | Diseased pig | 2 |  |
| 214 | DP1513 |  | AZM | CLI | DOX | ERY | FFC | GEN | TET | TIA |  |  |  |  |  |  |  |  |  | 107 | Diseased pig | 2 |  |
| 215 | DP1514 |  | AZM | CLI | DOX | ERY | FFC | GEN | TET | TIA |  |  |  |  |  |  |  |  |  | 107 | Diseased pig | 2 |  |
| 216 | DP1521 |  | AZM | CLI | DOX | ERY | FFC | GEN | TET | TIA | NOR | SXT |  |  |  |  |  |  |  | 108 * | Diseased pig | 2 |  |

| **Supplementary data** | | | | |  |  |  |  |  |  |  |  |  |  |  |  |  |  |  |  |  |  |  |
| --- | --- | --- | --- | --- | --- | --- | --- | --- | --- | --- | --- | --- | --- | --- | --- | --- | --- | --- | --- | --- | --- | --- | --- |
| **Table S1 (continued)** | | | | |  |  |  |  |  |  |  |  |  |  |  |  |  |  |  |  |  |  |  |
|  |  |  |  |  |  |  |  |  |  |  |  |  |  |  |  |  |  |  |  |  |  |  |  |
| **No.** | **Strain** |  | **AMR patterns** | | | | | | | | | | | | | | | | | **AMR pattern No.** | **Source** | **Serotype** |  |
| 217 | DP6020 |  | AZM | CHL | CLI | DOX | ERY | FFC | GEN | TET | TIA | CIP | ENR | NOR | LEV | SXT |  |  |  | 109 * | Diseased pig | AA |  |
| 218 | AP145 |  | AMP | CFL | PEN | CLI | TET | TIA | CIP | ENR | NOR | SXT |  |  |  |  |  |  |  | 110 * | Asymptomatic pig | AA |  |
| 219 | AP142 |  | AMP | CFL | PEN | CLI | DOX | GEN | TET | TIA | NOR | SXT |  |  |  |  |  |  |  | 111 * | Asymptomatic pig | AA |  |
| 220 | AP141 |  | AMP | CFL | PEN | CLI | DOX | GEN | TET | TIA | CIP | ENR | NOR | SXT |  |  |  |  |  | 112 * | Asymptomatic pig | AA |  |
| 221 | AP154 |  | AMP | CFL | PEN | CLI | DOX | GEN | TET | TIA | CIP | ENR | NOR | LEV | SXT |  |  |  |  | 113 * | Asymptomatic pig | AA |  |
| 222 | AP143 |  | AMP | CFL | PEN | CLI | DOX | TET | TIA | LEV | SXT |  |  |  |  |  |  |  |  | 114 * | Asymptomatic pig | AA |  |
| 223 | AP150 |  | AMP | CFL | PEN | CHL | CLI | GEN | TET | TIA | CIP | ENR | NOR | SXT |  |  |  |  |  | 115 * | Asymptomatic pig | AA |  |
| 224 | AP149 |  | AMP | CFL | PEN | AZM | CLI | ERY | TET | TIA | SXT |  |  |  |  |  |  |  |  | 116 * | Asymptomatic pig | AA |  |
| 225 | AP144 |  | AMP | CFL | PEN | AZM | CLI | ERY | TET | TIA | SXT |  |  |  |  |  |  |  |  | 116 * | Asymptomatic pig | AA |  |
| 226 | AP061 |  | AMP | CFL | PEN | AZM | CLI | ERY | TET | TIA | CIP | ENR | NOR | SXT |  |  |  |  |  | 117 * | Asymptomatic pig | 22 |  |
| 227 | AP146 |  | AMP | CFL | PEN | AZM | CLI | DOX | ERY | TET | TIA |  |  |  |  |  |  |  |  | 118 | Asymptomatic pig | AA |  |
| 228 | AP148 |  | AMP | CFL | PEN | AZM | CLI | DOX | ERY | TET | TIA | NOR | SXT |  |  |  |  |  |  | 119 * | Asymptomatic pig | AA |  |
| 229 | AP147 |  | AMP | CFL | PEN | AZM | CLI | DOX | ERY | TET | TIA | LEV | SXT |  |  |  |  |  |  | 120 * | Asymptomatic pig | AA |  |
| 230 | AP065 |  | AMP | CFL | PEN | AZM | CLI | DOX | ERY | TET | TIA | CIP | NOR | SXT |  |  |  |  |  | 121 * | Asymptomatic pig | 9 |  |
| 231 | AP062 |  | AMP | CFL | PEN | AZM | CLI | DOX | ERY | TET | TIA | CIP | ENR | NOR | SXT |  |  |  |  | 122 * | Asymptomatic pig | 29 |  |
| 232 | AP156 |  | AMP | CFL | PEN | AZM | CLI | DOX | ERY | TET | TIA | CIP | ENR | NOR | SXT |  |  |  |  | 122 * | Asymptomatic pig | AA |  |
| 233 | AP157 |  | AMP | CFL | PEN | AZM | CLI | DOX | ERY | TET | TIA | CIP | ENR | NOR | SXT |  |  |  |  | 122 * | Asymptomatic pig | AA |  |
| 234 | AP069 |  | AMP | CFL | PEN | AZM | CLI | DOX | ERY | TET | TIA | CIP | ENR | NOR | LEV | SXT |  |  |  | 123 * | Asymptomatic pig | 22 |  |
| 235 | AP070 |  | AMP | CFL | PEN | AZM | CLI | DOX | ERY | TET | TIA | CIP | ENR | NOR | LEV | SXT |  |  |  | 123 * | Asymptomatic pig | 22 |  |
| 236 | AP133 |  | AMP | CFL | PEN | AZM | CLI | DOX | ERY | TET | TIA | CIP | ENR | NOR | LEV | SXT |  |  |  | 123 * | Asymptomatic pig | AA |  |
| 237 | AP159 |  | AMP | CFL | PEN | AZM | CLI | DOX | GEN | TET | TIA | CIP | ENR | NOR | LEV | SXT |  |  |  | 123 * | Asymptomatic pig | AA |  |
| 238 | AP153 |  | AMP | CFL | PEN | AZM | CLI | DOX | ERY | GEN | TET | TIA | SXT |  |  |  |  |  |  | 124 * | Asymptomatic pig | AA |  |
| 239 | AP151 |  | AMP | CFL | PEN | AZM | CLI | DOX | ERY | GEN | TET | TIA | NOR | SXT |  |  |  |  |  | 125 * | Asymptomatic pig | AA |  |
| 240 | AP185 |  | AMP | CFL | PEN | AZM | CLI | DOX | ERY | GEN | TET | TIA | NOR | LEV | SXT |  |  |  |  | 126 * | Asymptomatic pig | NT |  |
| 241 | AP068 |  | AMP | CFL | PEN | AZM | CLI | DOX | ERY | GEN | TET | TIA | CIP | ENR | NOR | SXT |  |  |  | 127 * | Asymptomatic pig | 29 |  |
| 242 | AP155 |  | AMP | CFL | PEN | AZM | CLI | DOX | ERY | GEN | TET | TIA | CIP | ENR | NOR | SXT |  |  |  | 127 * | Asymptomatic pig | AA |  |
| 243 | AP187 |  | AMP | CFL | PEN | AZM | CLI | DOX | ERY | GEN | TET | TIA | CIP | ENR | NOR | SXT |  |  |  | 127 * | Asymptomatic pig | NT |  |
| 244 | AP188 |  | AMP | CFL | PEN | AZM | CLI | DOX | ERY | FFC | GEN | TET | TIA | CIP | ENR | NOR | SXT |  |  | 128 * | Asymptomatic pig | NT |  |
| 245 | AP189 |  | AMP | CFL | PEN | AZM | CLI | DOX | ERY | FFC | GEN | TET | TIA | CIP | ENR | NOR | LEV | SXT |  | 129 * | Asymptomatic pig | NT |  |
| 246 | AP186 |  | AMP | CFL | PEN | AZM | CHL | CLI | DOX | ERY | TET | TIA | NOR | SXT |  |  |  |  |  | 130 * | Asymptomatic pig | NT |  |
| 247 | AP063 |  | AMP | CFL | PEN | AZM | CHL | CLI | DOX | ERY | TET | TIA | NOR | LEV | SXT |  |  |  |  | 131 * | Asymptomatic pig | 24 |  |
| 248 | AP067 |  | AMP | CFL | PEN | AZM | CHL | CLI | DOX | ERY | TET | TIA | CIP | ENR | NOR |  |  |  |  | 132 * | Asymptomatic pig | 29 |  |
| 249 | AP066 |  | AMP | CFL | PEN | AZM | CHL | CLI | DOX | ERY | GEN | TET | TIA |  |  |  |  |  |  | 133 | Asymptomatic pig | 9 |  |
| 250 | AP064 |  | AMP | CFL | PEN | AZM | CHL | CLI | DOX | ERY | GEN | TET | TIA | NOR |  |  |  |  |  | 134 * | Asymptomatic pig | 22 |  |
| 251 | AP071 |  | AMP | CFL | PEN | AZM | CHL | CLI | DOX | ERY | FFC | GEN | TET | TIA | CIP | ENR | NOR | SXT |  | 135 * | Asymptomatic pig | 30 |  |
| 252 | AP160 |  | AMP | CFL | PEN | AZM | CHL | CLI | DOX | ERY | FFC | GEN | TET | TIA | CIP | ENR | NOR | SXT |  | 135 * | Asymptomatic pig | AA |  |
| 253 | DP6022 |  | AMP | CFL | PEN | AZM | CHL | CLI | DOX | ERY | FFC | GEN | TET | TIA | CIP | ENR | NOR | LEV | SXT | 136 * | Diseased pig | 22 |  |

| **Supplementary data** | | | | |  |  |  |  |  |  |  |  |  |  |  |  |  |  |  |  |  |  |  |
| --- | --- | --- | --- | --- | --- | --- | --- | --- | --- | --- | --- | --- | --- | --- | --- | --- | --- | --- | --- | --- | --- | --- | --- |
| **Table S1 (continued)** | | | | |  |  |  |  |  |  |  |  |  |  |  |  |  |  |  |  |  |  |  |
|  |  |  |  |  |  |  |  |  |  |  |  |  |  |  |  |  |  |  |  |  |  |  |  |
| **No.** | **Strain** |  | **AMR patterns** | | | | | | | | | | | | | | | | | **AMR pattern No.** | **Source** | **Serotype** |  |
| 254 | DP6023 |  | AMP | CFL | PEN | AZM | CHL | CLI | DOX | ERY | FFC | GEN | TET | TIA | CIP | ENR | NOR | LEV | SXT | 136 * | Diseased pig | 22 |  |
| 255 | AP072 |  | AMP | CFL | CTX | PEN | AZM | CLI | DOX | ERY | GEN | TET | TIA | CIP | ENR | NOR | SXT |  |  | 137 * | Asymptomatic pig | 22 |  |
| 256 | AP158 |  | AMP | CFL | CTX | PEN | AZM | CHL | CLI | DOX | ERY | FFC | TET | TIA | SXT |  |  |  |  | 138 * | Asymptomatic pig | AA |  |
| 257 | DP6018 |  | AMP | CFL | CTX | CTF | PEN | VAN | CLI | DOX | GEN | TET |  |  |  |  |  |  |  | 139 | Diseased pig | 25 |  |
| 258 | DP6019 |  | AMP | CFL | CTX | CTF | PEN | AZM | CHL | CLI | DOX | ERY | TET | TIA | SXT |  |  |  |  | 140 | Diseased pig | NT |  |
| 259 | AP129 |  | AMP | CFL | CLI | DOX | TET | TIA | SXT |  |  |  |  |  |  |  |  |  |  | 141 * | Asymptomatic pig | AA |  |
| 260 | AP130 |  | AMP | CFL | AZM | CLI | DOX | ERY | TET | TIA | CIP | ENR | NOR | SXT |  |  |  |  |  | 142 * | Asymptomatic pig | AA |  |
| 261 | AP060 |  | AMP | CFL | AZM | CLI | DOX | ERY | GEN | TET | TIA | NOR | LEV | SXT |  |  |  |  |  | 143 * | Asymptomatic pig | 22 |  |
| 262 | AP057 |  | AMP | CFL | AZM | CLI | DOX | ERY | TET | NOR | SXT |  |  |  |  |  |  |  |  | 144 * | Asymptomatic pig | 22 |  |
|  |  |  |  |  |  |  |  |  |  |  |  |  |  |  |  |  |  |  |  |  |  |  |  |
