# Supplementary material for: Antimicrobial susceptibility of Streptococcus suis isolated from diseased pigs, asymptomatic pigs, and human patients in Thailand
Source: BMC Vet Res. 2019 Jan 3;15:5. doi: 10.1186/s12917-018-1732-5 (PMC6318959; doi:10.1186/s12917-018-1732-5)
Supplement: Supplementary file 6 — Table S3. Antimicrobial susceptibility of Thai serotype 2 Streptococcus suis. Human patients (27 strains) and pigs (32 strains), including asymptomatic (7 strains) and diseased pigs (25 strains). AMP: ampicillin, AZM: azithromycin, CTX: cefotaxime, CTF: ceftiofur, CFL: cephalexin, CHL: chloramphenicol, CIP: ciprofloxacin, CLI: clindamycin, DOX: doxycycline, ENR: enrofloxacin, ERY: erythromycin, FFC: florfenicol, GEN: gentamicin, LEV: levofloxacin, NOR: norfloxacin, PEN: penicillin G, SXT: sulfamethoxazole/trimethoprim, TET: tetracyclin, TIA: tiamulin, VAN: vancomycin. S: susceptible; I: intermediate; R: resistant. The asterisk indicates statistical significance with P-value < 0.05. (DOC 3450 kb) [file 12917_2018_1732_MOESM6_ESM.doc]

**Table S2**:

| **Antibiotic drugs** | **Diseased pigs** | | | | | | **P-value** |
| --- | --- | --- | --- | --- | --- | --- | --- |
|  | **Year: 2006-2007**  **23 strains** | | | **Year 2012-2015**  **23 strains** | | |  |
|  | **S** | **I** | **R** | **S** | **I** | **R** |  |
| AMP | 18 (78.3%) | 1 (4.3%) | 4 (17.4%) | 22 (95.7%) | 1 (4.3%) | 0 (0%) | 0.111 |
| CFL | 12 (52.2%) | 1 (4.3%) | 10 (43.5%) | 22 (95.7%) | 0 (0%) | 1 (4.3%) | 0.004^*^ |
| CTX | 16 (69.6%) | 2 (8.7%) | 5 (21.7%) | 22 (95.7%) | 0 (0%) | 1 (4.3%) | 0.060 |
| CTF | 17 (74.0%) | 3 (13.0%) | 3 (13.0%) | 22 (95.7%) | 0 (0%) | 1 (4.3%) | 0.098 |
| PEN | 12 (52.2%) | 7 (30.4%) | 4 (17.4%) | 21 (91.3%) | 1 (4.3%) | 1 (4.3%) | 0.013^*^ |
| VAN | 19 (82.7%) | 1 (4.3%) | 3 (13.0%) | 23 (100%) | 0 (0%) | 0 (0%) | 0.112 |
| AZM | 9 (39.1%) | 0 (0%) | 14 (60.9%) | 0 (0%) | 0 (0%) | 23 (100%) | 0.003^*^ |
| CHL | 17 (74.0%) | 1 (4.3%) | 5 (21.7%) | 20 (87%) | 3 (13%) | 0 (0%) | 0.044^*^ |
| CLI | 5 (21.7%) | 0 (0%) | 18 (78.3%) | 0 (0%) | 0 (0%) | 23 (100%) | 0.058 |
| DOX | 2 (8.7%) | 2 (8.7%) | 19 (82.6%) | 0 (0%) | 0 (0%) | 23 (100%) | 0.112 |
| ERY | 9 (39.1%) | 1 (4.3%) | 13 (56.6%) | 0 (0%) | 0 (0%) | 23 (100%) | 0.002^*^ |
| FFC | 19 (82.6%) | 0 (0%) | 4 (17.4%) | 17 (73.9%) | 0 (0%) | 6 (26.1%) | 0.721 |
| GEN | 7 (30.4%) | 5 (21.8%) | 11 (47.8%) | 4 (17.4%) | 4 (17.4%) | 15 (65.2%) | 0.462 |
| TET | 3 (13.0%) | 2 (8.7%) | 18 (78.3%) | 0 (0%) | 0 (0%) | 23 (100%) | 0.061 |
| TIA | 3 (13.0%) | 3 (13.0%) | 17 (74.0%) | 1 (4.3%) | 2 (8.7%) | 20 (87%) | 0.486 |
| CIP | 11 (47.8%) | 4 (17.4%) | 8 (34.8%) | 21 (91.3%) | 1 (4.3%) | 1 (4.3%) | 0.006^*^ |
| ENR | 10 (43.5%) | 5 (21.7%) | 8 (34.8%) | 13 (56.5%) | 10 (43.5%) | 0 (0%) | 0.007^*^ |
| NOR | 5 (21.7%) | 8 (34.8%) | 10 (43.5%) | 23 (100%) | 0 (0%) | 0 (0%) | 0.061 |
| LEV | 18 (78.3%) | 1 (4.3%) | 4 (17.4%) | 8 (34.8%) | 5 (21.7%) | 10 (43.5%) | 0.500 |
| SXT | 8 (34.8%) | 4 (17.4%) | 11 (47.8%) | 10 (43.5%) | 2 (8.7%) | 11 (47.8%) | 0.641 |
