# Supplementary material for: Antimicrobial susceptibility of Streptococcus suis isolated from diseased pigs, asymptomatic pigs, and human patients in Thailand
Source: BMC Vet Res. 2019 Jan 3;15:5. doi: 10.1186/s12917-018-1732-5 (PMC6318959; doi:10.1186/s12917-018-1732-5)
Supplement: Supplementary file 7 — Figure S4. Heatmap illustrates susceptibility, i.e. susceptible, intermediate, and resistant, of serotype 2 Streptococcus suis, isolated from human patients and pigs, towards testing antibiotic drugs. The isolated bacteria were clustered based on host, i.e. human patients (n = 27) and pigs (n = 32) including asymptomatic and diseased pigs. Associations between source of isolation and susceptibility of each antibiotic drug were analyzed using Pearson’s Chi-square dependent test. The asterisk indicates that null hypothesis of the Chi-square test was rejected (P-value < 0.05), suggesting a significant association. (DOC 3449 kb) [file 12917_2018_1732_MOESM7_ESM.doc]

**Table S3**:

| **Antibiotic drugs** | **Human patients**  **27 strains** | | | **Pigs**  **32 strains** | | | **P-value** |
| --- | --- | --- | --- | --- | --- | --- | --- |
|  | **S** | **I** | **R** | **S** | **I** | **R** |  |
| AMP | 27 (100%) | 0 (0%) | 0 (0%) | 31 (96.9%) | 1 (3.1%) | 0 (0%) | 1.000 |
| CFL | 27 (100%) | 0 (0%) | 0 (0%) | 31 (96.9%) | 0 (0%) | 1 (3.1%) | 1.000 |
| CTX | 27 (100%) | 0 (0%) | 0 (0%) | 31 (96.9%) | 0 (0%) | 1 (3.1%) | 1.000 |
| CTF | 27 (100%) | 0 (0%) | 0 (0%) | 31 (96.9%) | 0 (0%) | 1 (3.1%) | 1.000 |
| PEN | 27 (100%) | 0 (0%) | 0 (0%) | 30 (91.3%) | 1 (3.1%) | 1 (3.1%) | 0.418 |
| VAN | 5 (18.5%) | 0 (0%) | 22 (81.5%) | 32 (100%) | 0 (0%) | 0 (0%) | 0.515 |
| AZM | 24 (88.9%) | 3 (11.1%) | 0 (0%) | 0 (0%) | 0 (0%) | 32 (100%) | 0.038^*^ |
| CHL | 5 (18.5%) | 0 (0%) | 22 (81.5%) | 29 (90.6%) | 3 (9.4%) | 0 (0%) | 1.000 |
| CLI | 0 (0%) | 0 (0%) | 27 (100%) | 0 (0%) | 0 (0%) | 32 (100%) | 0.038^*^ |
| DOX | 4 (14.8%) | 4 (14.8%) | 19 (70.4%) | 0 (0%) | 0 (0%) | 32 (100%) | 0.515 |
| ERY | 27 (100%) | 0 (0%) | 0 (0%) | 0 (0%) | 1 (3.1%) | 31 (96.9%) | 0.016^*^ |
| FFC | 22 (81.5%) | 3 (11.1%) | 2 (7.4%) | 26 (81.2%) | 0 (0%) | 6 (18.8%) | 0.036^*^ |
| GEN | 27 (100%) | 0 (0%) | 0 (0%) | 11 (34.4%) | 5 (15.6%) | 16 (50%) | 0.001^*^ |
| TET | 0 (0%) | 23 (85.2%) | 4 (14.8%) | 0 (0%) | 0 (0%) | 32 (100%) | 0.515 |
| TIA | 20 (74.1%) | 5 (18.5%) | 2 (7.4%) | 1 (3.1%) | 7 (21.9%) | 24 (75%) | < 0.001^*^ |
| CIP | 15 (55.6%) | 12 (44.4%) | 0 (0%) | 27 (84.4%) | 2 (6.2%) | 3 (9.4%) | 0.347 |
| ENR | 5 (18.5%) | 19 (70.4%) | 8 (11.1%) | 17 (53.1%) | 15 (46.9%) | 0 (0%) | 1.000 |
| NOR | 27 (100%) | 0 (0%) | 0 (0%) | 10 (31.2%) | 8 (25%) | 14 (43.8%) | 0.002^*^ |
| LEV | 27 (100%) | 0 (0%) | 0 (0%) | 32 (100%) | 0 (0%) | 0 (0%) | 0.515 |
| SXT | 27 (100%) | 0 (0%) | 0 (0%) | 19 (59.4%) | 2 (6.2%) | 11 (34.4%) | 0.001^*^ |
