# Supplementary material for: Antimicrobial susceptibility of Streptococcus suis isolated from diseased pigs, asymptomatic pigs, and human patients in Thailand
Source: BMC Vet Res. 2019 Jan 3;15:5. doi: 10.1186/s12917-018-1732-5 (PMC6318959; doi:10.1186/s12917-018-1732-5)
Supplement: Supplementary file 8 — Table S4. Source of Streptococcus suis isolated strains and numbers of strains used in this study. Thai S. suis strains used in this study were isolated during 2006–2007, except 23 strains of serotype 2 S. suis obtained from central regions #2 (Nakhon Pathom) were isolated during 2012–2015. Non-serotype 2 S. suis isolated from diseased pigs includes serotype 1 (1 strain), 14 (1 strain), 16 (1 strain), 22 (2 strains), 23 (1 strain), 25 (1 strain), and 34 (1 strain). Non-serotype 2 S. suis isolated from asymptomatic pigs includes serotypes 1 (1 strain), 3 (5 strains), 5 (3strains), 7 (2 strains), 9 (7 strains), 12 (2 strains), 15 (1 strain), 16 (5 strains), 19 (2 strains), 21 (1 strain), 22 (17 strains), 24 (2strains), 25 (1 strain), 27 (1 strain), 28 (2 strains), 29(4 strains), 30 (6 strains), and 34 (3 strains). (DOC 3934 kb) [file 12917_2018_1732_MOESM8_ESM.doc]

**Supplementary data**

**Table S4:**

| **Thai *Streptococcus suis* isolated strains** | | **Serotype 2** | **Non-serotype 2** | **Autoagglutinating** | **Non-typable** |
| --- | --- | --- | --- | --- | --- |
| **Human patients**  **(27 strains)** | Epidemic: Northern regions (Phayao 15 strains and other 4 strains) | 19 | 0 | 0 | 0 |
|  | Sporadic: Northern regions (Phayao 7 strains and Phrae 1 strain) | 8 | 0 | 0 | 0 |
| **Diseased pigs**  **(46 strains)** | Northern regions (Phaoyao 11 strains) | 0 | 1 | 3 | 7 |
|  | Central regions #1 (Nakhon Pathom 6 strains)  Central regions #2 (Nakhon Pathom 23 strains) | 2  23 | 4  0 | 0  0 | 0  0 |
|  | Southern regions (Nakhon Si Thammarat 6 strains) | 0 | 3 | 0 | 3 |
| **Asymptomatic pigs**  **(189 strains)** | Northern regions (Phaoyao 189 strains) | 7 | 65 | 88 | 29 |
