# Supplementary material for: Antimicrobial susceptibility of Streptococcus suis isolated from diseased pigs, asymptomatic pigs, and human patients in Thailand
Source: BMC Vet Res. 2019 Jan 3;15:5. doi: 10.1186/s12917-018-1732-5 (PMC6318959; doi:10.1186/s12917-018-1732-5)
Supplement: Supplementary file 9 — Table S5. Inhibition zone diameter and zone interpretation of antibiotic drugs. Antibiotic drugs used in this study were classified into four different modes of inhibition including cell-wall synthesis inhibitors (6 drugs), protein synthesis inhibitors (9 drugs), DNA synthesis inhibitors (4 drugs), and antimetabolite (1 drug). The zone of inhibition was interpreted as susceptible (S), intermediate (I), or resistant (R), according to a standard protocol of Clinical & Laboratory Standards Institute (CLSI), European Committee on Antimicrobial Susceptibility Testing (EUCAST) and Ref. [20]. The interpretation according to EUCAST and CLSI 2013 was used for veterinary practice, described in EUCAST and CLSI-potency Neo-Sensitabs™ User’s Guide 2013, rev. date 11-04-2013 [30]. (DOC 3973 kb) [file 12917_2018_1732_MOESM9_ESM.doc]

**Supplementary data**

**Table S5:**

| **Antibiotic drugs** | | **Amount**  **per disk** | **Interpretation zone diameter (mm)** | | | **References** |
| --- | --- | --- | --- | --- | --- | --- |
|  |  |  | **S** | **I** | **R** |  |
| **Cell-wall synthesis** | Ampicillin (AMP) | 10 μg | ≥ 24 | 23-17 | ≤ 16 | EUCAST and CLSI 2013 [30] |
|  | Cephalexin (CFL) | 30 μg | ≥ 20 | 19-17 | ≤ 16 | EUCAST and CLSI 2013 [30] |
|  | Cefotaxime (CTX) | 30 μg | ≥ 24 | 22-23 | ≤ 21 | CLSI 2016 for *Streptococcus* spp. Viridans group [29] |
|  | Ceftiofur (CTF) | 30 μg | ≥ 21 | 18-20 | ≤ 17 | Soares TCS., et al. 2014 [20] |
|  | Penicillin G (PEN) | 10 units | ≥ 26 | 25-13 | ≤ 12 | EUCAST and CLSI 2013 [30] |
|  | Vancomycin (VAN) | 30 μg | ≥ 17 | 16-15 | ≤ 14 | EUCAST and CLSI 2013 [30] |
| **Protein synthesis** | Azithromycin (AZM) | 15 μg | ≥ 18 | 14-17 | ≤ 13 | CLSI 2016 for *Streptococcus* *pneumoniae* [29] |
|  | Chloramphenicol (CHL) | 30 μg | ≥ 21 | 18-20 | ≤ 17 | CLSI 2016 for *Streptococcus* spp. Viridans group [29] |
|  | Clindamycin (CLI) | 2 μg | ≥ 19 | 16-18 | ≤ 15 | CLSI 2016 for *Streptococcus* *pneumoniae* [29] |
|  | Doxycycline (DOX) | 30 μg | ≥ 28 | 25-27 | ≤ 24 | CLSI 2016 for *Streptococcus* *pneumoniae* [29] |
|  | Erythromycin (ERY) | 15 μg | ≥ 21 | 16-20 | ≤ 15 | CLSI 2016 for *Streptococcus* *pneumoniae* [29] |
|  | Florfenicol (FFC) | 30 μg | ≥ 22 | 19-21 | ≤ 18 | Soares TCS., et al. 2014 [20] |
|  | Gentamicin (GEN) | 10 μg | ≥ 16 | 13-15 | ≤ 12 | EUCAST and CLSI 2013 [30] |
|  | Tetracycline (TET) | 30 μg | ≥ 28 | 25-27 | ≤ 24 | CLSI 2016 for *Streptococcus* *pneumoniae* [29] |
|  | Tiamulin (TIA) | 30 μg | ≥ 25 | 19-24 | ≤ 18 | EUCAST and CLSI 2013 [30] |
| **DNA synthesis** | Ciprofloxacin (CIP) | 5 μg | ≥ 21 | 16-20 | ≤ 15 | Soares TCS., et al. 2014 [20] |
|  | Enrofloxacin (ENR) | 5 μg | ≥ 23 | 19-22 | ≤ 18 | Soares TCS., et al. 2014 [20] |
|  | Norfloxacin (NOR) | 10 μg | ≥ 17 | 13-16 | ≤ 12 | Soares TCS., et al. 2014 [20] |
|  | Levofloxacin (LEV) | 5 μg | ≥ 17 | 14-16 | ≤ 13 | CLSI 2016 for *Streptococcus* *pneumoniae* [29] |
| **Antimetabolite** | Sulfamethoxazole/  Trimethoprim (SXT) | 23.75/1.25 μg | ≥ 19 | 16-18 | ≤ 15 | CLSI 2016 for *Streptococcus* *pneumoniae* [29] |
